# Mutational analysis in *Corynebacterium stationis* MFS transporters for improving nucleotide bioproduction

**DOI:** 10.1007/s00253-024-13080-y

**Published:** 2024-03-04

**Authors:** Keita Kinose, Keiko Shinoda, Tomoyuki Konishi, Hisashi Kawasaki

**Affiliations:** 1 Agro-Biotechnology Research Center, Graduate School of Agriculture and Life Sciences, The University of Tokyo, Tokyo, Japan; 2 Nagahama Institute for Biochemical Science, Oriental Yeast Co., Ltd., Nagahama, Shiga Japan; 3 Collaborative Research Institute for Innovative Microbiology, The University of Tokyo, Tokyo, Japan; 4 Present Address: Research Organization of Information and Systems, The Institute of Statistical Mathematics, Tachikawa, Japan

**Keywords:** *Corynebacteriumstationis*, MFS transporter, Nucleotide transport, Microbial cell factory

## Abstract

**Abstract:**

Product secretion from an engineered cell can be advantageous for microbial cell factories. Extensive work on nucleotide manufacturing, one of the most successful microbial fermentation processes, has enabled *Corynebacterium stationis* to transport nucleotides outside the cell by random mutagenesis; however, the underlying mechanism has not been elucidated, hindering its applications in transporter engineering. Herein, we report the nucleotide-exporting major facilitator superfamily (MFS) transporter from the *C. stationis* genome and its hyperactive mutation at the G64 residue. Structural estimation and molecular dynamics simulations suggested that the activity of this transporter improved via two mechanisms: (1) enhancing interactions between transmembrane helices through the conserved “RxxQG” motif along with substrate binding and (2) trapping substrate-interacting residue for easier release from the cavity. Our results provide novel insights into how MFS transporters change their conformation from inward- to outward-facing states upon substrate binding to facilitate efflux and can contribute to the development of rational design approaches for efflux improvements in microbial cell factories.

**Keypoints:**

*• An MFS transporter from C. stationis genome and its mutation at residue G64 were assessed*

*• It enhanced the transporter activity by strengthening transmembrane helix interactions and trapped substrate-interacting residues*

*• Our results contribute to rational design approach development for efflux improvement*

**Graphical Abstract:**

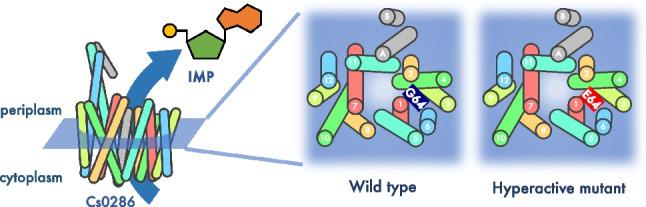

**Supplementary Information:**

The online version contains supplementary material available at 10.1007/s00253-024-13080-y.

## Introduction

Microbial cell factories and fermentation processes are becoming more important in the sustainable manufacturing of valuable compounds, and advances in synthetic biology and metabolic engineering are contributing to meeting their demand (Nielsen et al. 2022). However, optimizing the metabolic pathway of microbial cells is sometimes inadequate for target production. Accumulated targets may inhibit upstream enzymes to reduce the rate of synthesis, may reach the reaction equilibrium or degraded by intracellular enzymes to limit yield, and may cause cytotoxicity to lower titer. Therefore, ensuring efficient export of target products is important for their production (López et al. 2022; Lv et al. 2022; van der Hoek and Borodina 2020).

In this context, transporter engineering broadly focuses on target production. In addition to appropriate specific transporters (Hu et al. 2018; Steiger et al. 2019), transporters with a broad spectrum of substrate specificity—mechanosensitive channels (Kawasaki and Martinac 2020) and multidrug transporters (Wang et al. 2013; Sugita and Koketsu 2022)—are utilized for bioproduction. The latter transporters have advantages in a wide range of applications; therefore, further enhancement of secretion efficiency is desired. Mutations in a mechanosensitive channel MscCG cause constant glutamate secretion in *Corynebacterium glutamicum* and are utilized in the glutamate fermentation process, which is one of the most successful bioproduction processes (Nakamura et al. 2007). However, to our knowledge, mutations that enhance activity in any other cases have not been found so far.

Inosine monophosphate (IMP), which has a strong umami taste and is widely commercialized as a food additive, is produced by a fermentation process using *Corynebacterium stationis* (formerly known as *Corynebacterium* *ammoniagenes*). Random mutagenesis enabled IMP secretion in this species (Teshiba and Furuya 1982; Park et al. 2006). Literature has shown that in a bred *C. stationis* strain, the ability to export IMP is dependent on an “energy-generating system,” which is inhibited by electron transport chain inhibitors (Teshiba and Furuya 1984). This implies the possible involvement of ATP- or proton motive force (PMF)-dependent transporters in IMP secretion. However, no attempts have been made to investigate the corresponding mutations of this phenotype. Investigating this would provide a new clue for the improvement of bioproduction by efflux enhancements.

We focused on this by searching for nucleic acid efflux transporters and identifying mutations that result in their functional improvement. We collected IMP- or xanthosine monophosphate (XMP)-producing *C. stationis* strains, which are bred stepwise for commercial fermentation (Park et al. 2006, 2009; Kim et al. 2004; Kwag et al. 2008; Jinman et al. 2013), and read their genomes to investigate their mutational history. We found that five transporter genes were convergently mutated in two independently bred strains, and when expressed in the IMP-producing *E. coli*, one of the mutated genes had a significant ability to enhance its IMP accumulation. Among the two mutations found in the IMP-exporting major facilitator superfamily transporter (MFS), G64E substitution was correlated with its hyperactivity. Molecular dynamics simulation suggested that E64 has greater interaction with conserved motif B among drug-H^+^ antiporters.

The results we obtained offer insights into shared multidrug transporter mechanisms among bacteria and provide clues for rational mutagenesis of efflux improvement in microbial cell factories.

## Materials and methods

### Bacterial strains

*C. stationis* ATCC6872 was purchased from American Type Culture Collection (ATCC). KCCM10340, KCCM10448, KCCM10530, KCCM10972, and KCCM10610 were provided by the Korean Culture Center of Microorganisms (KCCM). These strains were used for genome analysis and IMP/XMP-producing analyses (Fig. S1). *E. coli* K-12 strain JM109 was used as a cloning host for plasmid construction. *E. coli* K-12 strain W3110 was used as the wild-type (WT) strain. *E. coli* I-9*ushAaphA*/pMWKQ (Kakehi et al. 2007) was provided by Ajinomoto Co., Inc. and was used to obtain an IMP-accumulating strain via *mscL* knockout. For the analyses shown in Figs. 2, 3 and 5, and Fig. S3, an empty pET-16 vector was co-transformed to enhance *lacI* expression to facilitate stable growth under non-induction conditions.

### Plasmid construction

All primers used in this study are listed in Table S4. The scheme for plasmid construction is described in detail below. PCR was performed using the KOD One PCR mix (TOYOBO, Osaka, Japan), and the coding sequences of the constructed plasmids were verified using Sanger sequencing.

For pMAN-∆mscL, the DNA fragment containing each 1.5 kb upstream and downstream of the *mscL* gene was artificially synthesized by Genscript Co., Ltd. (Piscataway, NJ, USA). The upstream and downstream fragments were cloned into pMAN997 plasmid (Matsui et al. 2001) using *Hind*III and *BamH*I restriction enzymes (Takara bio, Shiga, Japan) and a DNA ligase kit (Ligation high, TOYOBO).

For transporter expression plasmids, genes of interest were amplified using PCR on WT *C. stationis* ATCC6872, IMP producer KCCM10610, and XMP producer KCCM10340 using primer numbers 1 and 2 for *cs0286*^WT^ and *cs0286*^T43I^, numbers 2 and 3 for *cs0286*^V2L G64E^, numbers 4 and 5 for *cs0510*, numbers 6 and 7 for *cs0916*, numbers 8 and 9 for *cs0966*, and numbers 10 and 11 for *cs2429* (Table S4). Each transporter gene fragment was cloned into the *Eco*RI-digested pSTV29 vector using the homemade SLiCE reaction mix (Motohashi 2015).

Primers 12–21 were prepared for the site-directed mutagenesis of *cs0286*. PCR was performed using pSTV29-*cs0286*^WT^ as the template. Primers 12 and 13 were used for *cs0286*^V2L^; 14 and 15 for *cs0286*^G64E^; 16 and 17 for *cs0286*^R143A^; 18 and 19 for *cs0286*^Q146A^; and 20 and 21, for *cs0286*^N208A^. PCR fragments were cyclized using the SLiCE reaction. *cs0286*^G64E^ was used to prepare *cs0286*^G64E R143A^, *cs0286*^G64E Q146A^, and *cs0286*^G64E N208A^ in the same manner. For the experiment shown in Fig. S4, 6xHis-tag fused vectors were used. This vector was made with SLiCE reaction which concatenates synthesized 6xHis-tag fragment and non-tag vectors linearized by PCR with primers 26 and 27.

### mscL knockout

The I-9*ushAaphAmscL* strain was created by a previously reported method utilizing the temperature-sensitive replication origin of pMAN997 (Matsui et al. 2001). *E. coli* I-9*ushAaphA* was transformed with pMAN-∆mscL and cultured at 30 °C for the replication of *ori*^*ts*^. Genomically integrated transformants were selected by 42 °C culture under selective force with ampicillin. Transformants with a correctly inserted pMAN-∆mscL cassette in the genomic *mscL* locus were screened by PCR with primer sets numbers 22/23 and 24/25. The hit strain was cultured again at 30 °C, and single-colony clones were tested to determine whether they exhibited ampicillin resistance. The ampicillin-sensitive clones, which should have passed a secondary crossover-recombination event, were tested to check if the *mscL* locus was correctly disrupted by PCR with the number 22/25 primer set, followed by Sanger sequencing.

### Culture conditions

#### *C. stationis* IMP/XMP production analyses

Cells grown on CM2B plates (10 g/L polypeptone, 10 g/L yeast extract, 5 g/L NaCl, 10 μg/L biotin, 100 mg/L adenine, and 100 mg/L guanine) were inoculated into 20 mL seed medium (50 g/L glucose, 5 g/L polypeptone, 10 g/L yeast extract, 2.5 g/L NaCl, 100 mg/L adenine, and 100 mg/L guanine). After 24 h of cultivation at 31.5 °C, 3 mL of the seed culture was inoculated into 27 mL of the main medium (80 g/L glucose, 1 g/L sodium glutamate, 10 g/L NH_4_Cl, 25 g/L MgSO_4_·7H_2_O, 0.1 g/L CaCl_2_, 37 mg/L FeSO_4_·7H_2_O, 32 mg/L MnSO_4_·7H_2_O, 36 mg/L ZnSO_4_·7H_2_O, 8 mg/L CuSO_4_·5H_2_O, 23 mg/L L-cysteine, 24 mg/L alanine, 8 mg/L nicotinate, 45 μg/L biotin, 5 mg/L thiamine hydrochloride, 30 mg/L adenine, and 19 mL/L phosphoric acid [85%]). Cells were cultured at 31.5 °C and sampled chronologically.

#### *E. coli *IMP production analyses

**Fig. 1:**
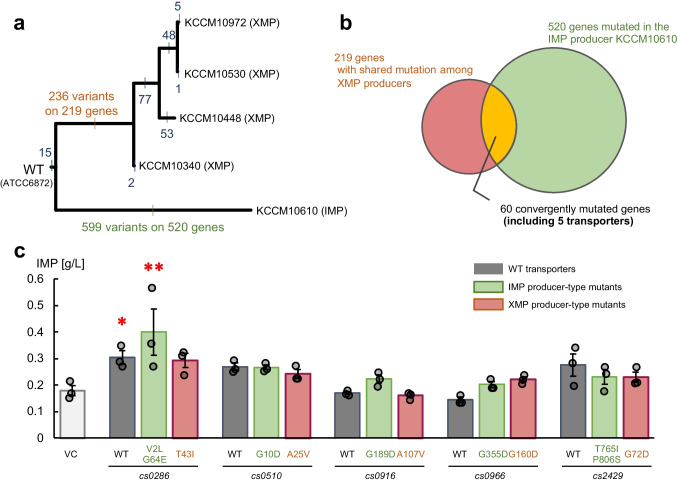
Identification of nucleotide transporters and their mutation for functional improvement from the *C. stationis* genome. **a** Mutational history of analyzed strains. Tree topology was constructed manually based on the patent information. The numbers on each branch indicate the number of nonsynonymous-mutational events on the genome in each breeding step.**b** Venn diagram of the designated gene sets: 219 genes with shared nonsynonymous mutations among XMP producers (KCCM10340, 10,448, 10,530, and 10,972) and 520 genes with nonsynonymous mutations in the IMP producer KCCM10610.** c** IMP production assay of transporter-overexpressing strains. The results for the wild-type (WT), IMP-producing strain-type mutants, and XMP-producing strain-type mutants are shown in this order, with the captions indicating the harbored mutations. The means of three replicates are shown as bars. Error bars represent the standard error of the mean. Circles represent the values of each replicate. *p < 0.05, **p < 0.01 vs. vector control as analyzed using Dunnett’s test

**Fig. 2:**
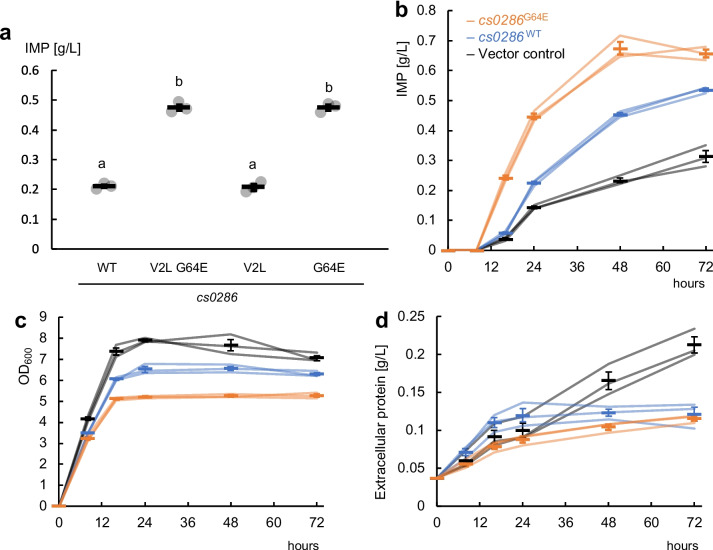
G64E mutation is responsible for enhanced inosine monophosphate (IMP) secretion from the transporter **a** IMP production assay of the strains overexpressing *cs0286* and its mutants. Bars show the mean of three independent experiments, and individual data are shown as circles. Error bars represent the standard error of the mean. Different letters indicate statistically significant differences as analyzed using Tukey’s honest significant difference test (*p* < 0.01). **b**–**d** Chronological changes in IMP-producing cultures. The IMP concentration in the media **b**, bacterial cell growth **c**, and protein enrichment in the culture **d** are shown. Each line indicates the result of three independent experiments, and the means ± standard errors are shown in bars

**Fig. 3:**
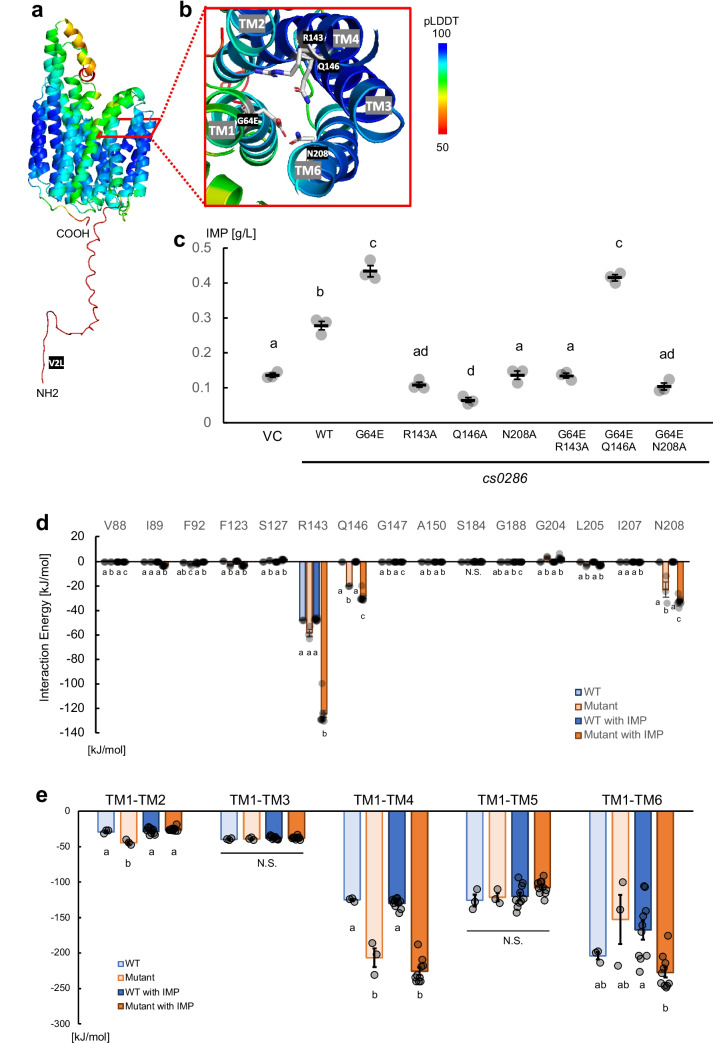
Glutamate at position 64 strengthens the interaction of TM1 with TM4 and TM6, affecting Cs0286 activity. **a** AlphaFold2-estimated structure of the Cs0286 V2L G64E mutant. **b** Magnified image of the E64 residue. The model is colored using the predicted local distance difference test (pLDDT) value, showing the confidence level of the conformation. **c** IMP production assay of strains overexpressing *cs0286* wild type (WT) and its mutants. VC represents the vector control. The bars represent the means ± standard errors of three independent experiments. Different letters indicate statistically significant differences as analyzed using Tukey’s honest significant difference test (*p* < 0.01). **d** Interaction energies of G64 or E64 with residues in other helices. The calculated interaction energies between G64 or E64 and residues on other helices within a 0.8-nm distance of G64 or E64 were averaged over 3 (without inosine monophosphate [IMP]) or 10 (with IMP) independent trajectories for the last 20 or 100 ns. The average values are indicated by bars. Error bars represent standard error of means. The circles represent the average values for each trajectory. Different letters in each group indicate statistically significant differences as analyzed using Tukey’s honest significant difference test (*p* < 0.01). N.S., not significant. **e** The calculated interaction energies between TM1 and other helices in the N lobe of Cs0286 were averaged over 3 (without IMP) or 10 (with IMP) independent trajectories for the last 20 or 100 ns. The average values are indicated by bars with the standard errors. The circles represent the average values for each trajectory. Different letters in each group indicate statistically significant differences as analyzed using Tukey’s honest significant difference test (*p* < 0.01). N.S., not significant

Cells grown on LB plates were inoculated into the modified MS medium composed of 40 g/L glucose, 1 g/L MgSO_4_·7 H_2_O, 16 g/L (NH_4_)_2_SO_4_, 1 g/L KH_2_PO_4_, 0.01 g/L FeSO_4_ ·7 H_2_O, 0.01 g/L MnSO_4_ 7H_2_O, 8 g/L yeast extract, and 30 g/L CaCO_3_ and cultivated at 37 °C (Fig. 1) or 30 °C (Figs. 2, 3, 5, and Fig. S2) for 24 h, unless otherwise noted. Growth was assessed by measuring the optical density at 600 nm. Purine nucleotides in the culture supernatant were separated by HPLC using a GS-220HQ column (Shodex, Tokyo, Japan) and 0.2 M NaPO buffer (pH 4.0) as the mobile phase and were detected spectrophotometrically by monitoring the absorbance at 254 nm. Protein levels in the growth media were quantified with Bradford’s method using the Bio-Rad Protein Assay Kit following the manufacturer’s protocol (Bio-Rad Laboratories, Hercules, CA, USA). All experiments were performed with more than three biological replicates.

### Calculation of metabolic kinetics

For the measurement of the intracellular and extracellular nucleotides, sampled cell cultures were freeze-thawed and heated at 85 °C for 5 min with intermittent vortexing before centrifugation. After heat extraction, extracts containing intracellular and extracellular nucleotides were measured as described above. Using data at 16 and 24 h, when cell growth ceased and IMP, inosine (Ino), and hypoxanthine (Hyp) content increased linearly, kinetic parameters *v*_1_, *v*_2_, and *v*_3_ were calculated as follows:

$$\begin{array}{l}{v}_{1}=\frac{\Delta {(\left[{\text{IMP}}\right]}_{{\text{in}}+{\text{out}}}+{\left[{\text{Ino}}\right]}_{{\text{in}}+{\text{out}}}+{\left[{\text{Hyp}}\right]}_{{\text{in}}+{\text{out}}})}{\Delta t} /{\text{average}}({{\text{OD}}}_{600}) \\ {v}_{2}=\frac{\Delta {\left[{\text{IMP}}\right]}_{{\text{out}}}}{\Delta t} /{\text{average}}({{\text{OD}}}_{600})\\ {v}_{3}=\frac{\Delta ({\left[{\text{Ino}}\right]}_{{\text{in}}+{\text{out}}}+{\left[{\text{Hyp}}\right]}_{{\text{in}}+{\text{out}}})}{\Delta t} /{\text{average}}({{\text{OD}}}_{600})\end{array}$$

### Protein extraction and Western blotting

Cells cultured for 24 h as described above were collected and resuspended in buffer (20 mM Tris–HCl, 300 mM NaCl, 3 mM MgSO_4_, 10% w/v glycerol, pH 8) to an OD_600_ of 10. Proteins were extracted by ultrasonication and concentrations were determined using the Pierce™ BCA Protein Assay Kit (Thermo Fisher Scientific, Waltham, MA, USA). Ten micrograms of lysate was loaded on the SuperSep™Ace 10–20% gradient polyacrylamide gels (Wako, Osaka, Japan) with the sample buffer solution with reducing reagent (Nacalai tesque, Kyoto, Japan). After SDS-PAGE, proteins were transferred to the membrane using iBlot®2 PVDF kit (Thermo Fisher Scientific) following the manufacturer’s protocol. The membranes were soaked in the blocking solution (Nacalai tesque) overnight at 4 °C, washed, and incubated in the His-tag antibody (Cat# MAB050, R&D systems, Minneapolis, MN, USA) at 1:1000 dilution for 3 h at room temperatures. After washing, membranes were incubated in the HRP-fused secondary antibody (Cat# 8738, CellSignaling technology, Danvers, MA, USA) at 1:5000 dilution for 3 h at room temperatures. After washing, bands were visualized with Immobilon® Forte western HRP substrate (Merck Millipore, Burlington, MA, USA).

### Whole genome sequencing

Genomic DNA from the strains of *C. stationis* ATCC6872, KCCM10340, KCCM10448, KCCM10530, KCCM10972, and KCCM10610 was isolated using the Wizard Genomic DNA Purification Kit (Promega, Madison, WI, USA). Genome sequence analysis was performed at Macrogen Co., Ltd. (Seoul, Korea) with ATCC6872 (WT) genomic DNA using the SMRT sequencing with the PacBio platform (Menlo Park, CA, USA) and Illumina sequencing (San Diego, CA, USA). PacBio reads were assembled using HGAP (v3.0), and errors in the draft assembly were corrected with Illumina reads (> 1000 × depth, 150 bp paired ends) using Pilon (v1.21). Gene annotation was performed using the Prokka pipeline (v1.12b).

Genomic DNA of KCCM10340, KCCM10448, KCCM10530, KCCM10972, and KCCM10610 was used for library preparation on an Illumina platform with 150 bp paired-end reads. Read depths exceeding 1000 × were acquired, and single-nucleotide polymorphisms (SNPs) were called using the ATCC6872 genome as a reference. BWA (v0.7.17), SAMTools, and SnpEff (v4.3t) were used for the mapping, variant calling, and annotation of SNPs, respectively. Among the SNPs, nonsynonymous mutations were filtered, and the inclusion relations of SNPs of each strain were counted using the R package VennDiagram (v1.7.3).

### Molecular dynamics (MD) simulations

The MD simulations were performed using GROMACS version 2020.6 (Abraham et al. 2015). The all-atom Fuji force field (Kamiya et al. 2020; Fujitani et al. 2009) was used for the proteins, lipids, and ions in TIP3P water (Jorgensen et al. 1986). All systems were hydrated with 150 mM NaCl electrolyte. The temperature was set to a constant (298 K) using a Nosé–Hoover thermostat (Nosé, 1984; Hoover 1985) with a coupling constant of 1.0. The pressure was set to 1 bar constant, using a semi-isotropic Berendsen barostat (Berendsen et al. 1984) or a Parrinello–Rahman barostat (Parrinello and Rahman 1981) with a coupling constant of 2.0 ps for both barostats. Electrostatic interactions were calculated using the particle mesh Ewald (PME) method (Darden et al. 1993) with a real-space cut-off of 1.0 nm. The Lennard–Jones interactions were calculated using the Lennard–Jones particle mesh Ewald (LJ-PME) method (Wennberg et al. 2015) with geometric approximations of the combination rules in reciprocal space. The Verlet cut-off scheme was used for the neighbor list. The linear constraint solver (LINCS) algorithm (Hess 2008) with a LINCS-order of six was used to constrain all the bonds. To remove the bond-angle degrees of freedom from hydrogen atoms, a virtual site model was adapted.

The initial coordinates of Cs0286 and its mutant were built using Alphafold2 (Jumper et al. 2021; Mirdita et al. 2022). The initial proteins were embedded in a pre-equilibrated *E. coli* model membrane (for 1 μs) consisting of six lipids (652 molecules of PMPE [1-palmitoyl-2-cis-9,10-methylene-hexadecanoic-acid-sn-glycero-3-phosphoethanolamine]; 181 molecules of PMPG [1-palmitoyl-2-cis-9,10-methylene-hexadecanoic-acid-sn-glycero-3-phosphoglycerol]; 40 molecules of PVPE [1-palmitoyl-2-vacenoyl-sn-glycero-3-phosphatidylethanolamine]; 68 molecules of HYPE [1-palmitoleoyl-2-cis-11,12-methylene-hexadecanoic-acid-sn-glycero-3-phosphoethanolamine]; 101 molecules of PMHP [1-palmitoyl-2-cis-11,12-methylene-hexadecanoic-acid-sn-glycero-3-phosphoethanolamine]; and 41 molecules of cardiolipin [1-(1-palmitoyl-2-cis-9,10-methylene-hexadecanoic-acid-sn-3-phosphatidyl)-3-(1-palmitoyl-2-oleoyl-sn-3-phosphatidyl)-sn-glycerol]) using LAMBADA (Schmidt and Kandt 2012). Overlapping lipids whose heavy atoms were within 0.12 nm of those of the protein were removed. In systems containing IMP, the initial position of IMP was set using the CDOCKER module in Discovery Studio 2020, placing it in the largest cavity within the protein. The position set was similar to the corresponding position of chloramphenicol (Cm) in the co-crystal structure of MdfA and Cm (PDB:4ZOW, Heng et al. 2015). System energy was minimized using alternating steepest-descent and conjugate gradient methods, followed by isothermal-isobaric ensemble equilibration using the semi-isotropic Berendsen barostat, with position restraints only on the heavy atoms of the protein for 30 ns with a time step of 2 fs. Then, unconstrained simulations were performed using a semi-isotropic Berendsen barostat, initially for 20 ns with a time step of 2 fs and later for 10 ns with a time step of 4 fs. Using the final structures as the initial structure, three unbiased simulations were carried out with a time step of 4 fs, using a semi-isotropic Parrinello–Rahman barostat with a different initial velocity that satisfied a Maxwell–Boltzmann distribution at 298 K. Production runs were performed for three 100-ns runs for the IMP-free system and ten 340-ns runs for the system with IMP. Interaction energies were calculated using the GROMACS gmx energy tool, and the trajectories of the last 20 ns (without IMP) or 100 ns (with IMP) were used.

### Statistical analyses

Statistical analyses were performed using R (v4.2.0) and its package multcomp (v1.4). For the data in Fig. 1c, Dunnett’s tests were performed. VC was set as a control. Tukey’s honest significant difference tests were performed for the data shown in Figs. 2a, 3c–e, 5a–c, and Fig. S2. Data were visualized using Microsoft Excel.

## Results

### Searching *C. stationis* for nucleotide transporters

First, we searched the genome of nucleotide-producing *C. stationis* for transporters that contribute to nucleic acid secretion owing to mutations introduced during random mutagenesis. One IMP-producing strain (KCCM10610; Park et al. 2006) and four stepwise-bred XMP-producing strains (KCCM10340; Kim et al. 2004, KCCM10448; Kwag et al. 2008, KCCM10530; Park et al. 2009, and KCCM10972; Jinman et al. 2013) were selected for the analyses so that their mutation history could be traced (Fig. S1a). The WT and three strains were cultured for validation. KCCM10610 strain showed higher IMP accumulation than the WT strain (Fig. S1b). KCCM10972, KCCM10340, and the WT strain showed increased XMP accumulation, in this order, representing improvements via breeding (Fig. S1c). Notably, KCCM10340, the earliest XMP-fermentable strain bred, has significant XMP-exporting ability. We hypothesized that the nucleotide-exporting phenotype was acquired independently during the early breeding stages.

To elucidate the gene(s) responsible for this phenotype, we analyzed the whole genomes of these strains. The newly determined genome sequence of the WT *C. stationis* ATCC6872 showed high similarity to a recently reported genome of the same strain by Liu et al. (2016) (Table S1). The SNPs in the genomes of the IMP- and XMP-producer strains were called using the newly assembled genome (Table S2). We focused on nonsynonymous mutations and estimated the breeding step at which the mutation events occurred, based on which strains shared SNPs (Fig. 1a). Among the 219 genes in which nonsynonymous mutations were shared among the XMP-producers, 60 were also mutated in the IMP-producer KCCM10610. Five of these genes encoded transporters (Fig. 1b, Table S3). Mutations in these transporters may improve IMP transport. We cloned the *cs0286*, *cs0510*, *cs0916*, *cs0966*, and *cs2429* transporter genes from WT *C. stationis* ATCC6872, the IMP-producer KCCM10610, and the XMP-producer KCCM10340.

The *E. coli* strain I-9ushAaphA/pMWKQ, which expresses a desensitized mutant of the key enzyme PurF^K326Q^ in the background of the 11-gene deletion mutant (∆*purF* ∆*purA* ∆*deoD* ∆*purR* ∆*add* ∆*edd* ∆*yicP* ∆*pgi* ∆*xapA* ∆*ushA* ∆*aphA*), has a high IMP-synthesizing ability, but lacks adequate IMP-exporting capacity (Matsui et al. 2001; Shimaoka et al. 2005; Shimaoka et al. 2006; Shimaoka et al. 2007; Kakehi et al. 2007); hence, extracellular IMP levels can indicate the transportation of IMP across the membrane. We modified this strain with an additional knockout of *mscL*, the gene encoding a large conductive mechanosensitive channel with a pore size large enough to transport IMP, to ensure minimal IMP leakage. Candidate transporters were expressed in *E. coli* I-9ushAaphAmscL/pMWKQ to determine whether they could transport IMP (Fig. 1c). Compared to that with the vector control, the presence of plasmids harboring the WT *cs0286* led to a significantly higher IMP accumulation in the media at 48 h (*p* < 0.05), suggesting transport activity. Mutants of the XMP-producing strain type *cs0966* and IMP-producing strain types *cs0286* and *cs0916* led to a higher IMP accumulation than did the WT of these genes. These results suggest that IMP efflux is mediated by mutations in multiple transporter genes. Among the genes tested, *cs0286* and its IMP-producer-type mutant *cs0286*^V2L G64E^ showed significant effects. This was confirmed by a fermentation experiment under optimized conditions, performed with low temperatures and overexpression of *lacI* to suppress leakage of expression in non-induction conditions to ensure stable growth (Fig. S2). We performed a more detailed analysis of the effect of *cs0286* and its mutant, which may be a particularly important contributor to IMP accumulation in the *C. stationis* strain KCCM10610.

### Cs0286 is an MFS transporter that exports IMP

The *cs0286* gene encodes a 14-spanner transmembrane protein comprising 549 amino acids (Fig. S3a). It has some sequence similarities to known MFS transporters, including Motif B, which is partially conserved among some drug:H^+^ antiporters (Fig. S3b). *cs0286* was grouped into the drug:H^+^ antiporter 2 (DHA2)-type MFS based on its phylogenetic relationship with *E. coli* homologs (Fig. S3c). It likely exports IMP coupled with H^+^ import using PMF, corroborating a previous study finding that the electron transport chain plays a central role in IMP export (Teshiba and Furuya 1984).

Next, we investigated whether V2L or G64E mutations intensified the effect of *cs0286* by expressing single mutants of this gene. We found that the G64E mutant, but not the V2L mutant, led to greater IMP accumulation in the medium than did the WT *cs0286* (Fig. 2a). The G64E mutation did not affect protein expression level (Fig. S4). These findings suggest that the G64E mutation enhanced the transporter activity of Cs0286 protein.

We measured intracellular and extracellular IMP and its degradant level in chronological samples (Fig. S5b–d). Intracellular IMP is rapidly degraded to inosine (Ino) and then to hypoxanthine (Hyp), both of which readily permeate membranes. Since the I-9ushAaphA/pMWKQ strain lacks periplasmic 5′ nucleotidase activity (Kakehi et al. 2007), the extracellular accumulation of IMP is equal to its secretion rate (*v*_2_). From this model shown in Fig. S5a, we calculated total purine synthesis rate (*v*_1_) and IMP degradation rate (*v*_3_). The result was consistent with that the *cs0286* expression did not alter nucleotide production but promoted IMP export, and its G64E mutation enhanced the effect (Fig. S5e).

The presence of extracellular IMP in non-transporter-expressing lines could be explained by cell lysis. In the non-transporter-expressing control, extracellular IMP levels increased with protein accumulation in the culture supernatant, which could be attributed to cell lysis during the stationary phase. The expression of *cs0286* and its mutation enhanced IMP accumulation without accumulation of proteins in the media (Fig. 2b–d), possibly suggesting that transporters help export overproduced IMP to prevent cell lysis, leading to increased production.

### G64E mutation enhances IMP efflux via Cs0286 by an unknown mechanism

To address how this mutation affects transporter mechanisms, we predicted the structure of the Cs0286 protein and its mutant form using AlphaFold2 (Jumper et al. 2021; Mirdita et al. 2022) (Fig. 3a). The predicted structure of WT and mutant proteins did not show substantial differences (5.4 Å at RMSD). These overall structures were superimposable upon the solved structures of MFS transporters (Brawley et al. 2022; Kumar et al. 2021; Wisedchaisri et al. 2014; Fig. S6). Residue 64 in the first transmembrane helix (TM1) was predicted to face the outside of the central cavity and was unlikely to interact with any substrate molecule (Fig. 3b), implying that this mutation did not directly change the affinity to the substrate, but interacted with amino acid residues on other helices.

Next, we performed all-atom MD simulations of Cs0286 with and without IMP using the predicted structure as the initial structure. A highly precise FUJI force field (Fujitani et al. 2009; Kamiya et al. 2020) was used to simulate the behavior of proteins in bacterial membranes accurately. We calculated the interaction energies between G/E64 and other residues on different helices (Fig. 3d). In the WT protein, G64 in TM1 interacted mainly with R143 in TM4, whereas in the mutant protein, E64 interacted with R143 and Q146 in TM4 and N208 in TM6. In the Cs0286 mutant, the interaction between the R143 and E64 residues increased in the presence of IMP. Based on these results, we hypothesized that the R143, Q146, and N208 residues contribute to the hyperactivity of the Cs0286 G64E mutant.

The R143 and Q146 residues are conserved as an “RxxQG” motif (Motif B) among some MFS transporters (Fig. S3b). Based on the structure of MFS-type drug:H^+^ antiporters, such as *E. coli* MdfA, Motif B is thought to be involved in the PMF-dependent activity of the transporter. The positive electrostatic field of the basic arginine-containing Motif B is speculated to promote the deprotonation of nearby acidic amino acid residues into an inward-open conformation (Heng et al. 2015; Zhang et al. 2015). However, this explanation cannot be applied to Cs0286 as the WT transporter, unlike other PMF-dependent transporters, such as MdfA, lacks an acidic amino acid residue close to Motif B. We constructed mutants with R143A, Q146A, and N208A substitutions to investigate the contribution of these residues to Cs0286 activity. All mutants expressed on a level with WT protein, whereas showed decreased IMP accumulation (Figs. 3c and S4), suggesting that these residues are required for the activity of Cs0286, regardless of the presence or absence of nearby acidic amino acid residues.

### Helix–helix interaction is important for Cs0286 activity

To understand the contribution of these residues better, we calculated the interaction energies between TM1 and other helices in the N lobe (i.e., TM2–TM6) in the simulation. In the WT, TM1 had notable interactions with TM4, TM5, and TM6 (Fig. 3e). The TM1–TM4 interaction was largely dependent on the G64 main chain and R143 residues (Fig. S7a). This finding explains that the decreased interaction between TM1 and TM4 was involved in the diminished transporter activity observed when residue R143 was substituted with alanine. The G64E mutation significantly enhanced the interaction between TM1 and TM4 (Fig. 3e), indicating a relationship between TM1–TM4 interaction and the activity of Cs0286. This helix–helix interaction would be necessary for the transporter activity.

In addition to E64 and R143, the Q65 and Q146 residues contributed to this interaction in the mutant Cs0286 (Fig. S7b). Because the G64E mutation restored reduced IMP export in the Q146A mutant, but not in the R143A and N208A mutants (Fig. 3c), helix interactions with these residues likely play a dominant role in the rigidity of both the mutant and WT Cs0286.

IMP binding slightly increased the TM1–TM4 interaction, with increased interaction for E64-R143 as compared to that for G64-R143 (Fig. 3d, e). The interaction between TM1 and TM6 fluctuated in the multiple simulation runs; however, in the presence of IMP, this interaction was relatively stronger in the mutant than in the WT, with the contribution of E64 and N208 residues (Fig. 3d, e). IMP binding is thus likely to stabilize the conformation of the N lobe.

### Effect of G64E on the IMP-interacting residue

**Fig. 4:**
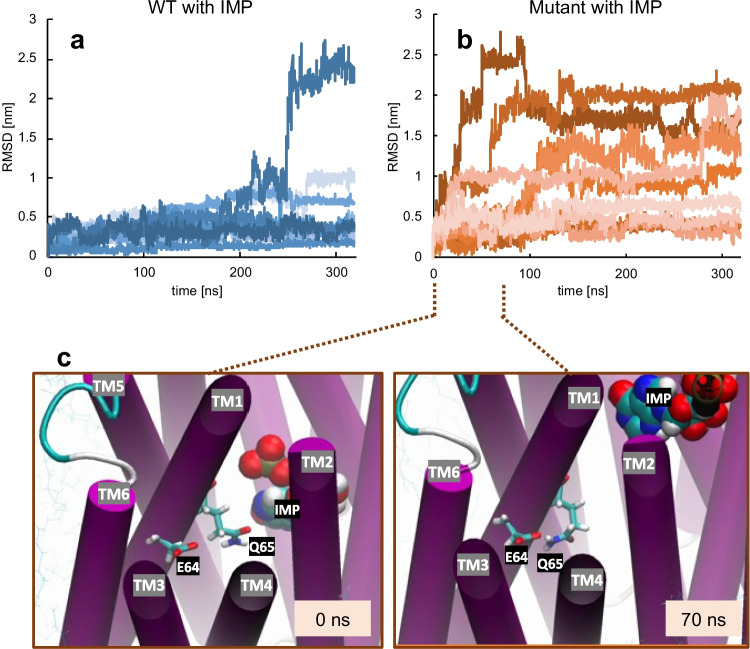
Inosine monophosphate (IMP) easily moves in the extracellular direction as shown by the molecular dynamic simulation of the Cs0286 mutant. **a**, **b** Time evolution of the root mean square deviations (RMSDs) of IMP during the simulation, showing the distance migrated from their initial positions of IMP for the wild type (WT; a) and mutant **b**. The RMSDs were calculated from trajectories fitted to the backbone of protein structure at *t* = 0. **c** Snapshots of the Q65–IMP interacting state (left) and non-interacting state (right) (see Movie S1)

In a simulation with WT Cs0286 and IMP, IMP interacted with several residues. In particular, Q65, which is located next to G/E64, was suggested to have a relatively strong interaction with IMP (Fig. S8). In the ten 340-ns simulation runs, IMP moved from the site in WT Cs0286 to the periplasm in only one run. By contrast, IMP moved more easily in the simulation of the mutant with IMP (Figs. 4a, b and S9). At the moment of IMP release, in most simulation runs in which IMP detached, the E64 residue appeared to support IMP release by attracting the Q65 residue that was interacting with IMP (Fig. 4c, Movie S1). This finding implies that in addition to changes in the TM1–TM4 and TM1–TM6 interactions for conformational changes, trapping the Q65 residue to prevent interaction with IMP for substrate release may also contribute to Cs0286^G64E^ hyperactivity.

**Fig. 5:**
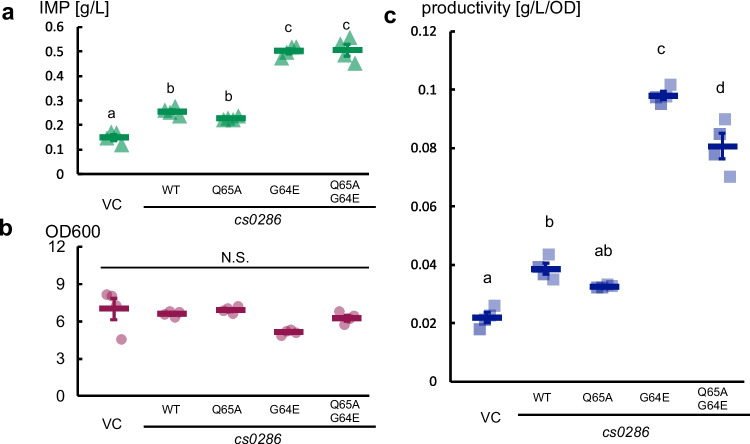
The Q65A mutation affects the transporter activity of Cs0286. Inosine monophosphate (IMP) production assay of overexpressing strains of *cs0286* wild type (WT) and its mutants. IMP titer (**a**), bacterial growth measured at OD_600_ (**b**), and productivity per cell (**c**) are shown. VC represents vector control. The bars represent the means ± standard errors of four independent experiments. Different letters in each panel indicate statistically significant differences as analyzed using Tukey’s honest significant difference test (*p* < 0.01). N.S., not significant

We further determined the contribution of the Q65 residue to IMP efflux in *E. coli*. The Q65A and G64E/Q65A mutations did not cause any significant change in protein expression levels (Fig. S4) and IMP accumulation as compared to those seen with the WT and G64E mutants, respectively (Fig. 5a). Because the bacteria expressing the G64E mutant grew slower than did the WT and Q65A mutant (Fig. 5b), we assessed whether IMP accumulation per cell was altered by mutation at the Q65 residue. The increase in IMP accumulation by expression of the G64E mutant was partially inhibited by alanine substitution at the Q65 residue (Fig. 5c). These data supported the hypothesis that the Q65 residue is involved in the mechanism of hyperactivation caused by G64E substitution in Cs0286.

## Discussion

In this study, we identified the MFS-type nucleotide transporter, Cs0286, in the *C. stationis* genome. The IMP-producing strain KCCM10610 harbors the hyperactive mutation G64E in Cs0286. The effect of the G64E mutation on Cs0286 activity may be explained in two ways: strengthening the TM1–TM4 and TM1–TM6 interaction, along with the binding of IMP, and trapping of the Q65 residue to ensure easier IMP release.

**Fig. 6:**
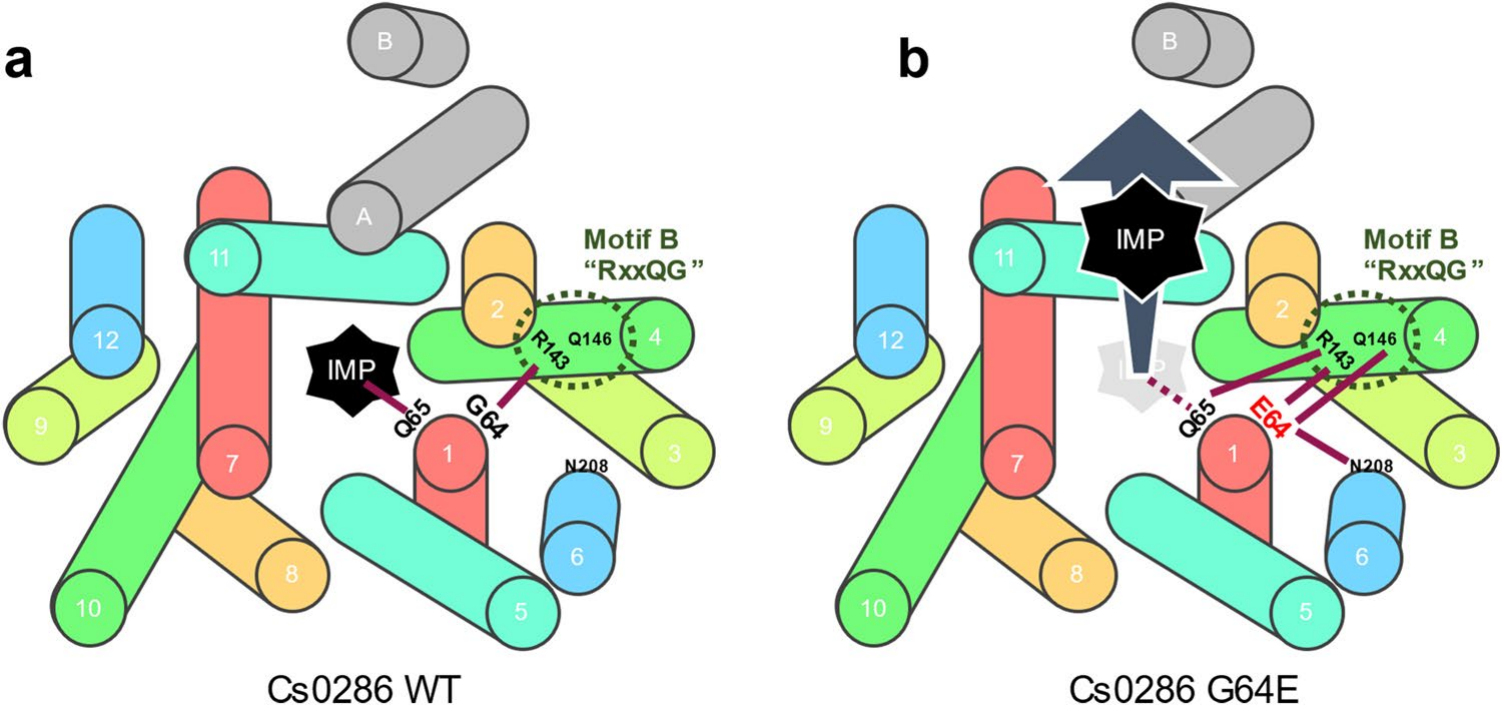
Proposed mechanism of IMP release from Cs0286 and its hyperactive mutant. Periplasmic views of the wild-type (**a**) and mutant (**b**) Cs0286 transporters are shown. Dark red lines indicate the interacting residues. The dotted line in panel (**b**) indicates that the interaction between IMP and Q65 is weaker than that in panel (**a**) due to the interaction with the E64 residue in the mutant

MFS transporters have two structurally similar lobes (N- and C-lobes), and it is believed that when transporting substrate molecules, these two rigid lobes rearrange symmetrically around the central substrate-binding site from inward- to outward- conformation or vice versa (Drew et al. 2021; Quistgaard et al. 2016). It is conceivable that the interactions between residues in the lobes support the movements of each helix as a bundle. The enhancement of these interactions caused by G64E mutation possibly resulted in the easier movement of these lobes as a bundle and higher IMP efflux (Fig. 6).

The predicted structures we used are likely in the occluded conformation. The binding of sugar to MFS-type sugar porters, which are H^+^ symporters involved in sugar uptake, has been proposed to stabilize the occluded conformation; thus, the binding and dissociation of sugar are the driving forces underlying the conformational change of the transporter (Madej et al. 2014). Although we could not simulate the conformational change, IMP binding to the G64E mutant also resulted in a higher interaction between helices of N-lobes. More stable intermediate structures for substrate binding may also contribute to the increased activity of this mutant.

It is worth noting that our findings are based on estimated structures and not observations of actual conformational changes in the simulation. Thus, questions remain regarding how exactly the strengthened interactions between transmembrane helices affect the conformational rearrangement during the transport, and which residue(s) besides Motif B are involved remains unknown. Longer MD simulations or structural analyses focusing on the intermediate state are needed to address these questions. Answering them may make it possible to modify transporters of other substrates for microbial cell factories to facilitate the efflux of products.

Additionally, it should be mentioned that we did not address the physiological role of this transporter in *C. stationis*. Kwon et al. (2021) recently reported that the knockout of this transporter (named ImpE2 in the literature) diminished IMP secretive phenotype in *C. stationis* strain CJI0323. Moreover, mutations in both the V2 and G64 residues of Cs0286, together with a mutation in the upstream transcription factor Cs0287 (named ImpE1 in the literature), are necessary for IMP secretion in this IMP-fermentative strain. If the V2 residue is unlikely to play any role in transporter function, mutations around the transcriptional start site and upstream transcriptional factors may disrupt regulation of the expression of *cs0286. C. stationis* may regulate the gene expression depending on environmental changes.

Although we used IMP production as an indicator in this study, natural substrates of the transporter remain unknown. *C. stationis* was isolated from the intestinal tract of infants based on ammonia production from urea (Cooke and Keith 1927). The growth of animal fecal bacteria relies on vitamins and enzyme cofactors from other members of the gut microbiota (Das et al. 2019; Sharma et al. 2019; Soto-Martin et al. 2020). Host animals are also dependent on this cross-feeding (LeBlanc et al. 2013). Cs0286 may facilitate the exchange of similar nucleotides or vitamins in the gut environment. Understanding Cs0286’s substrate specificity and expression regulation in *C. stationis* can shed light on the metabolic cooperation between the microbiome and host. By clarifying its substrate specificity, Cs0286 may be adapted to increase the production of nutrients that are deficient due to such alterations of the intestinal environment in microbial cell factories.

## Supplementary Information

Below is the link to the electronic supplementary material.

Supplementary file1 (PDF 1173 KB)

## Data Availability

All sequence data, including plasmid maps, genome assemblies, and SNP information, are available upon reasonable request to the corresponding author. All data have been uploaded to Figshare and are available at https://doi.org/10.6084/m9.figshare.25158419.

## References

[CR1] Abraham MJ, Murtola T, Schulz R, Páll S, Smith JC, Hess B, Lindahl E (2015) GROMACS: high performance molecular simulations through multi-level parallelism from laptops to supercomputers. SoftwareX 1–2:19–25. 10.1016/j.softx.2015.06.001

[CR2] Berendsen HJC, Postma JPM, van Gunsteren WF, DiNola A, Haak JR (1984) Molecular dynamics with coupling to an external bath. J Chem Phys 81:3684–3690. 10.1063/1.448118

[CR3] Brawley DN, Sauer DB, Li J, Zheng X, Koide A, Jede G, Suwantthee T, Song J, Liu Z, Arora PS, Koide S, Torres VJ, Wang D, Traaseth NJ (2022) Structural basis for inhibition of the drug efflux pump NorA from *Staphylococcus aureus*. Nat Chem Biol 18:706–712. 10.1038/s41589-022-00994-9 35361990 10.1038/s41589-022-00994-9 PMC9246859

[CR4] Cooke JV, Keith HR (1927) A type of urea-splitting bacterium found in the human intestinal tract. J Bacteriol 13(5):315–319. 10.1128/jb.13.5.315-319.1927 16559250 10.1128/jb.13.5.315-319.1927 PMC374939

[CR5] Darden T, York D, Pedersen L (1993) Particle mesh Ewald: an N⋅log(N) method for Ewald sums in large systems. J Chem Phys 98:10089–10092. 10.1063/1.464397

[CR6] Das P, Babaei P, Nielsen J (2019) Metagenomic analysis of microbe-mediated vitamin metabolism in the human gut microbiome. BMC Genomics 20:208. 10.1186/s12864-019-5591-7 30866812 10.1186/s12864-019-5591-7 PMC6417177

[CR7] Drew D, North RA, Nagarathinam K, Tanabe M (2021) Structures and general transport mechanisms by the major facilitator superfamily (MFS). Chem Rev 121:5289–5335. 10.1021/acs.chemrev.0c00983 33886296 10.1021/acs.chemrev.0c00983 PMC8154325

[CR8] Fujitani H, Matsuura A, Sakai S, Sato H, Tanida Y (2009) High- level ab initio calculations to improve protein backbone dihedral parameters. J Chem Theory Comput 5:1155–1165. 10.1021/ct8005437 26609625 10.1021/ct8005437

[CR9] Heng J, Zhao Y, Liu M, Liu Y, Fan J, Wang X, Zhao Y, Zhang XC (2015) Substrate-bound structure of the E. coli multidrug resistance transporter MdfA. Cell Res 25:1060–1073. 10.1038/cr.2015.94 26238402 10.1038/cr.2015.94 PMC4559816

[CR10] Hess B (2008) P-LINCS: a parallel linear constraint solver for molecular simulation. J Chem Theory Comuput 4:116–122. 10.1021/ct700200b 10.1021/ct700200b 26619985

[CR11] Hoover WG (1985) Canonical dynamics: equilibrium phase-space distributions. Phys Rev A 31:1695–1697. 10.1103/physreva.31.1695 10.1103/physreva.31.1695 9895674

[CR12] Hu Y, Zu Z, Nielsen J, Siewers V (2018) Heterologous transporter expression for improved fatty alcohol secretion in yeast. Metab Eng 45:51–58. 10.1016/j.ymben.2017.11.008 29183749 10.1016/j.ymben.2017.11.008

[CR13] Iancu CV, Zamoon J, Woo SB, Aleshin A, Choe JY (2013) Crystal structure of a glucose/H+ symporter and its mechanism of action. Proc Natl Acad Sci USA 110:17862–17867. 10.1073/pnas.1311485110 24127585 10.1073/pnas.1311485110 PMC3816430

[CR14] Jinman C, Kim H, Oh Y, Park J (2013) Corynebacteria strain for enhancement of 5′-guanosine monophosphate productivity and a method of producing 5′-guanosine monophosphate using the same. U.S. Patent No. 8,530,200. Washington, DC: U.S. Patent and Trademark Office

[CR15] Jorgensen WL, Chandrasekhar J, Buckner JK, Madura JD (1986) Computer simulations of organic reactions in solution. Ann N Y Acad Sci 482:198–209. 10.1111/j.1749-6632.1986.tb20951.x 3471104 10.1111/j.1749-6632.1986.tb20951.x

[CR16] Jumper J, Evans R, Pritzel A, Green T, Figurnov M, Ronneberger O, Tunyasuvunakool K, Bates R, Žídek A, Potapenko A, Bridgland A, Meyer C, Kohl SAA, Ballard AJ, Cowie A, Romera-Paredes B, Nikolov S, Jain R, Adler J, Back T, Petersen S, Reiman D, Clancy E, Zielinski M, Steinegger M, Pacholska M, Berghammer T, Bodenstein S, Silver D, Vinyals O, Senior AW, Kavukcuoglu K, Kohli P, Hassabis D (2021) Highly accurate protein structure prediction with AlphaFold. Nature 596:583–589. 10.1038/s41586-021-03819-2 34265844 10.1038/s41586-021-03819-2 PMC8371605

[CR17] Kakehi M, Usuda Y, Tabira Y, Sugimoto S (2007) Complete deficiency of 5′-nucleotidase activity in *Escherichia coli* leads to loss of growth on purine nucleotides but not of their excretion. Microb Physiol 13:96–104. 10.1159/000103601 10.1159/000103601 17693717

[CR18] Kamiya N, Kayanuma M, Fujitani H, Shinoda K (2020) A new lipid force field (FUJI). J Chem Theory Comput 16(6):3664–3676. 10.1021/acs.jctc.9b01195 32384238 10.1021/acs.jctc.9b01195

[CR19] Kawasaki H, Martinac B (2020) Mechanosensitive channels of *Corynebacterium glutamicum* functioning as exporters of L-glutamate and other valuable metabolites. Curr Opn Chem Biol 59:77–83. 10.1016/j.cbpa.2020.05.005 10.1016/j.cbpa.2020.05.005 32650225

[CR20] Kim J, Kwag Y, Park J, Koh E, Oh Y, Chang J, Lee K, Sim J, Han J, Park Y (2004) Corynebacterium ammoniagenes KCCM 10340 for producing 5'-xanthylic acid. U.S. Patent No. 6,821,768. Washington, DC: U.S. Patent and Trademark Office

[CR21] Kumar S, Athreya A, Gulati A, Nair RM, Mahendran I, Ranjan R, Penmatsa A (2021) Structural basis of inhibition of a transporter from *Staphylococcus aureus*, NorC, through a single-domain camelid antibody. Commun Biol 4:836. 10.1038/s42003-021-02356-x 34226658 10.1038/s42003-021-02357-x PMC8257674

[CR22] Kwag Y, Oh K, Kim J, Oh Y, Sim J, Park Y, Chang, J (2008) Microorganism producing 5′-xanthylic acid. U.S. Patent No. 7,456,010. Washington, DC: U.S. Patent and Trademark Office

[CR23] Kwon J, Baek M, Lee J, Kwon N, Kim J, Rho J, Cho J (2021) Polypeptide and method of producing IMP using the same. U.S. Patent No. 11,180,754. Washington, DC: U.S. Patent and Trademark Office

[CR24] LeBlanc JG, Milani C, de Giori GS, Sesma F, van Sinderen D, Ventura M (2013) Bacteria as vitamin suppliers to their host: a gut microbiota perspective. Curr Opin Biotechnol 24:160–168. 10.1016/j.copbio.2012.08.005 22940212 10.1016/j.copbio.2012.08.005

[CR25] Liu Y, Yang J, Jiang Y, Yang S (2016) Complete genome sequence of nucleoside producing strain *Corynebacterium stationis* ATCC 6872. J Biotechnol 225:57–58. 10.1016/j.jbiotec.2016.03.026 26995608 10.1016/j.jbiotec.2016.03.026

[CR26] López JM, Duran L, Avalos JL (2022) Physiological limitations and opportunities in microbial metabolic engineering. Nat Rev Microbiol 20:35–48. 10.1038/s41579-021-00600-0 34341566 10.1038/s41579-021-00600-0

[CR27] Lv X, Xue H, Qin L, Li C (2022) Transporter engineering in microbial cell factory boosts biomanufacturing capacity. BioDes Res 2022:9871087. 10.34133/2022/9871087 10.34133/2022/9871087 PMC10521751 37850143

[CR28] Madej MG, Sun L, Yan N, Kaback HR (2014) Functional architecture of MFS D-glucose transporters. Proc Natl Acad Sci USA 111(7):E719–E727. 10.1073/pnas.1400336111 24550316 10.1073/pnas.1400336111 PMC3932877

[CR29] Matsui H, Kawasaki H, Shimaoka M, Kurahashi O (2001) Investigation of various genotype characteristics for inosine accumulation in *Escherichia coli* W3110. Biosci Biotechnol Biochem 65:570–578. 10.1271/bbb.65.570 11330670 10.1271/bbb.65.570

[CR30] Mirdita M, Schütze K, Moriwaki Y, Heo L, Ovchinnikov S, Steinegger M (2022) ColabFold: making protein folding accessible to all. Nat Methods 19:679–682. 10.1038/s41592-022-01488-1 35637307 10.1038/s41592-022-01488-1 PMC9184281

[CR31] Motohashi K (2015) A simple and efficient seamless DNA cloning method using SLiCE from *Escherichia coli* laboratory strains and its application to SLiP site-directed mutagenesis. BMC Biotechnol 15:47. 10.1186/s12896-015-0162-8 26037246 10.1186/s12896-015-0162-8 PMC4453199

[CR32] Nakamura J, Hirano S, Ito H, Wachi M (2007) Mutations of the *Corynebacterium glutamicum* NCgl1221 gene, encoding a mechanosensitive channel homolog, induce L-glutamic acid production. Appl Environ Microbiol 73:4491–4498. 10.1128/AEM.02446-06 17513583 10.1128/AEM.02446-06 PMC1932805

[CR33] Nielsen J, Tillegreen CB, Petranovic D (2022) Innovation trends in industrial biotechnology. Trends Biotechnol 40:1160–1172. 10.1016/j.tibtech.2022.03.007 35459568 10.1016/j.tibtech.2022.03.007

[CR34] Nosé S (1984) A molecular dynamics method for simulations in the canonical ensemble. MOl Phys 52:255–268. 10.1080/00268978400101201

[CR35] Park Y, Kim H, Chohi H, Lee J, Hwang S, Sim J, Kang T, Lee W (2006)Microorganism producing 5'-inosinic acid and production method of 5'-inosinic acid using the same. Korean Patent No. 100,588,577. Seoul: Korean Intellectual Property Office

[CR36] Park Y, Chang J, Lee J, Oh K, Kim J, Oh Y, Sim J (2009) Microorganism producing 5′-xanthylic acid. U.S. Patent No. 7,608,435. Washington, DC: U.S. Patent and Trademark Office

[CR37] Parrinello M, Rahman A (1981) Polymorphic transitions in single crystals: a new molecular dynamics method. J Appl Phys 52:7182–7190. 10.1063/1.328693

[CR38] Paulsen PA, Custódio TF, Pedersen BP (2019) Crystal structure of the plant symporter STP10 illuminates sugar uptake mechanism in monosaccharide transporter superfamily. Nat Commun 10:407. 10.1038/s41467-018-08176-9 30679446 10.1038/s41467-018-08176-9 PMC6345825

[CR39] Quistgaard EM, Löw C, Guettou F, Nordlund P (2016) Understanding transport by the major facilitator superfamily (MFS): structures pave the way. Nat Rev Mol Cell Biol 17:123–132. 10.1038/nrm.2015.25 26758938 10.1038/nrm.2015.25

[CR40] Remm S, de Vecchis D, Schöppe J, Hutter CAJ, Gonda I, Hohl M, Newstead S, Schäfer LV, Seeger MA (2023) Structural basis for triacylglyceride extraction from mycobacterial inner membrane by MFS transporter Rv1410. bioRxiv 2023.01.25.525346. 10.1101/2023.01.25.525346 10.1038/s41467-023-42073-0 PMC10576003 37833269

[CR41] Schmidt TH, Kandt C (2012) LAMBADA and InflateGRO2: efficient membrane alignment and insertion of membrane proteins for molecular dynamics simulations. J Chem Inf Model 52:2657–2669. 10.1021/ci3000453 22989154 10.1021/ci3000453

[CR42] Sharma V, Rodionov DA, Leyn SA, Tran D, Iablokov SN, Ding H, Peterson DA, Osterman AL, Peterson SN (2019) B-vitamin sharing promotes stability of gut microbial communities. Front Microbiol 10:1485. 10.3389/fmicb.2019.01485 31333610 10.3389/fmicb.2019.01485 PMC6615432

[CR43] Shimaoka M, Kawasaki H, Takenaka Y, Kurahashi O, Matsui H (2005) Effects of *edd* and *pgi* disruptions on inosine accumulation in *Escherichia coli*. Biosci Biotechnol Biochem 69:1248–1255. 10.1271/bbb.69.1248 16041126 10.1271/bbb.69.1248

[CR44] Shimaoka M, Takenaka Y, Mihara Y, Kurahashi O, Kawasaki H, Matsui H (2006) Effects of *xapA* and *guaA* disruption on inosine accumulation in *Escherichia coli*. Biosci Biotechnol Biochem 70:3069–3072. 10.1271/bbb.60398 17151449 10.1271/bbb.60398

[CR45] Shimaoka M, Takenaka Y, Kurahashi O, Kawasaki H, Matsui H (2007) Effect of amplification of desensitized *purF* and *prs* on inosine accumulation in *Escherichia coli*. J Biosci Bioeng 103:255–261. 10.1263/jbb.103.255 17434429 10.1263/jbb.103.255

[CR46] Soto-Martin EC, Warnke I, Farquharson FM, Christodoulou M, Horgan G, Derrien M, Faurie JM, Flint HJ, Duncan SH, Louis P (2020) Vitamin Biosynthesis by Human Gut Butyrate-Producing Bacteria and Cross-Feeding in Synthetic Microbial Communities mBio 11:e00886-e920. 10.1128/mBio.00886-20 32665271 10.1128/mBio.00886-20 PMC7360928

[CR47] Steiger MG, Rassinger A, Mattanovich D, Sauer M (2019) Engineering of the citrate exporter protein enables high citric acid production in *Aspergillus niger*. Metab Eng 52:224–231. 10.1016/j.ymben.2018.12.004 30553933 10.1016/j.ymben.2018.12.004

[CR48] Sugita T, Koketsu K (2022) Transporter engineering enables the efficient production of lacto-*N*-triose II and lacto-*N*-tetraose in *Escherichia coli*. J Agric Food Chem 70:5106–5114. 10.1021/acs.jafc.2c01369 35426313 10.1021/acs.jafc.2c01369

[CR49] Teshiba S, Furuya A (1982) Mechanisms of 5′-inosinic acid accumulation by permeability mutants of *Brevibacterium ammoniagenes.* I. Genetical improvement of 5′-IMP productivity of a permeability mutant of *B. ammoniagenes*. Agric Biol Chem 46:2257–2563. 10.1080/00021369.1982.10865429

[CR50] Teshiba S, Furuya A (1984) Mechanisms of 5′-inosinic acid accumulation by permeability mutants of *Brevibacterium ammoniagenes*. IV. Excretion mechanisms of 5′-IMP. Chem Biol Technol Agric 48:1311–1317. 10.1080/00021369.1984.10866311

[CR51] van der Hoek SA, Borodina I (2020) Transporter engineering in microbial cell factories: the ins, the outs, and the in-betweens. Curr Opin Biotech 66:186–194. 10.1016/j.copbio.2020.08.002 32927362 10.1016/j.copbio.2020.08.002 PMC7758712

[CR52] Wang J, Xiong Z, Li S, Wang Y (2013) Enhancing isoprenoid production through systematically assembling and modulating efflux pumps in *Escherichia coli*. Appl Microbiol Biotechnol 97:8057–8067. 10.1007/s00253-013-5062-z 23864262 10.1007/s00253-013-5062-z

[CR53] Wennberg CL, Murtola T, Páll S, Abraham MJ, Hess B, Lindahl E (2015) Direct-space corrections enable fast and accurate Lorentz-Berthelot combination rule Lennard-Jones lattice summation. J Chem Theory Comput 11:5737–5746. 10.1021/acs.jctc.5b00726 26587968 10.1021/acs.jctc.5b00726

[CR54] Wisedchaisri G, Park MS, Iadanza MG, Zheng H, Gonen T (2014) Proton-coupled sugar transport in the prototypical major facilitator superfamily protein XylE. Nat Commun 5:4521. 10.1038/ncomms5521 25088546 10.1038/ncomms5521 PMC4137407

[CR55] Zhang XC, Zhao Y, Heng J, Jiang D (2015) Energy coupling mechanisms of MFS transporters. Protein Sci 24:1560–1579. 10.1002/pro.2759 26234418 10.1002/pro.2759 PMC4594656

