# Supplementary material for: Mutational analysis in Corynebacterium stationis MFS transporters for improving nucleotide bioproduction
Source: Appl Microbiol Biotechnol. 2024 Mar 4;108:251. doi: 10.1007/s00253-024-13080-y (PMC10912292; doi:10.1007/s00253-024-13080-y)
Supplement: Supplementary file 1 [file 253_2024_13080_MOESM1_ESM.pdf]

Supplementary Information

Journal name: *Applied Microbiology and Biotechnology*
Title:
Mutational analysis in *Corynebacterium stationis* MFS transporters for improving nucleotide bioproduction

Authors:
Keita Kinose[1,2,†], Keiko Shinoda[1,3,†], Tomoyuki Konishi[1], and Hisashi Kawasaki[1,3,*]

Affiliations:
[1]Agro-Biotechnology Research Center, Graduate School of Agriculture and Life Sciences, The University of Tokyo, Tokyo, Japan
[2]Nagahama Institute for Biochemical Science, Oriental Yeast Co., Ltd., Shiga, Japan
[3]Collaborative Research Institute for Innovative Microbiology, The University of Tokyo, Tokyo, Japan
†These authors contributed equally to this work.
*Correspondence and requests for materials should be addressed to H.K. (email: ukawasaki@g.ecc.u-tokyo.ac.jp).

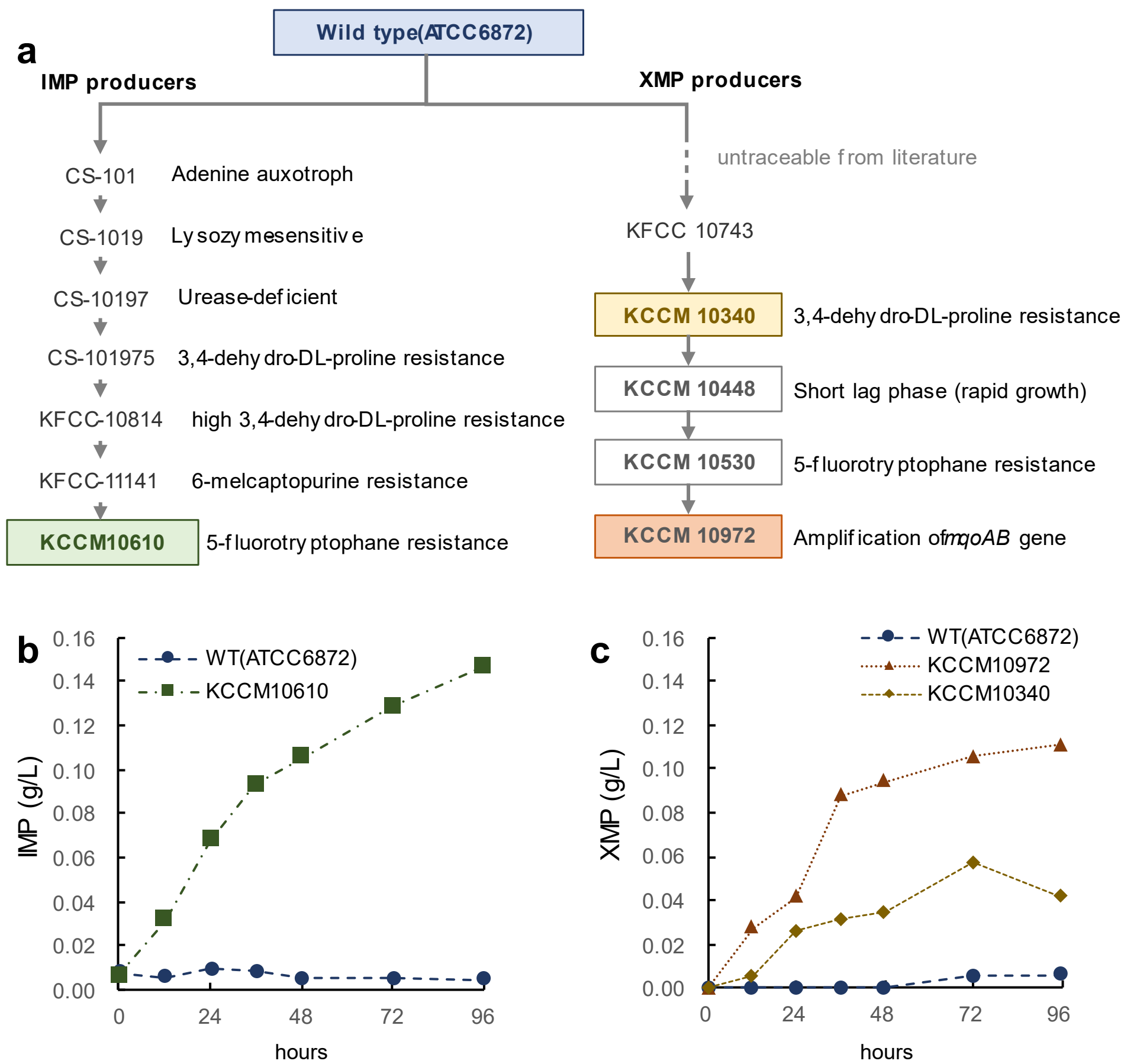

Fig. S1 Nucleotide secretion phenotype was acquired in the early stages of breeding.

(a) Selection history of nucleotide-fermenting *C. stationis*. Accession numbers of mutants and their selection criteria are shown. Boxed strains were used for genome analyses. The colored strains were assayed for nucleotide production to confirm their phenotypes.

(b) Inosine mononucleotide (IMP)-producing phenotypes of commercially mutated strains. Representative data from five independent analyses are shown.

(c) Xanthosine monophosphate (XMP)-producing phenotypes of commercially mutated strains. Representative data from three independent analyses are shown.

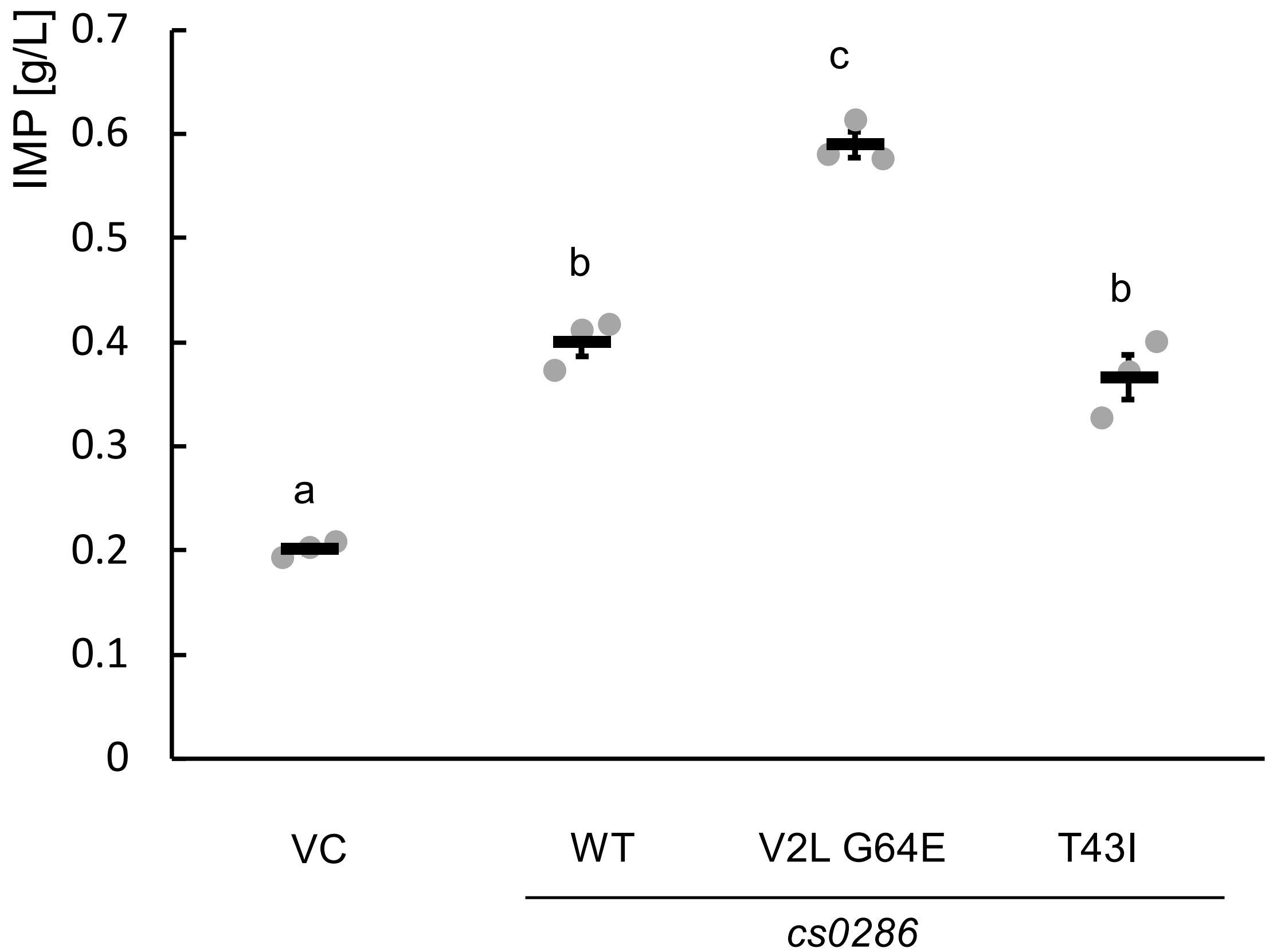

Fig. S2 Inosine mononucleotide (IMP) production assay of overexpressed strains of cs0286 and its mutants.

VC represents the vector control. The bars represent the means ± standard errors of three independent experiments. Different letters indicate statistically significant differences determined using Tukey's honest significant difference test ($p < 0.01$).

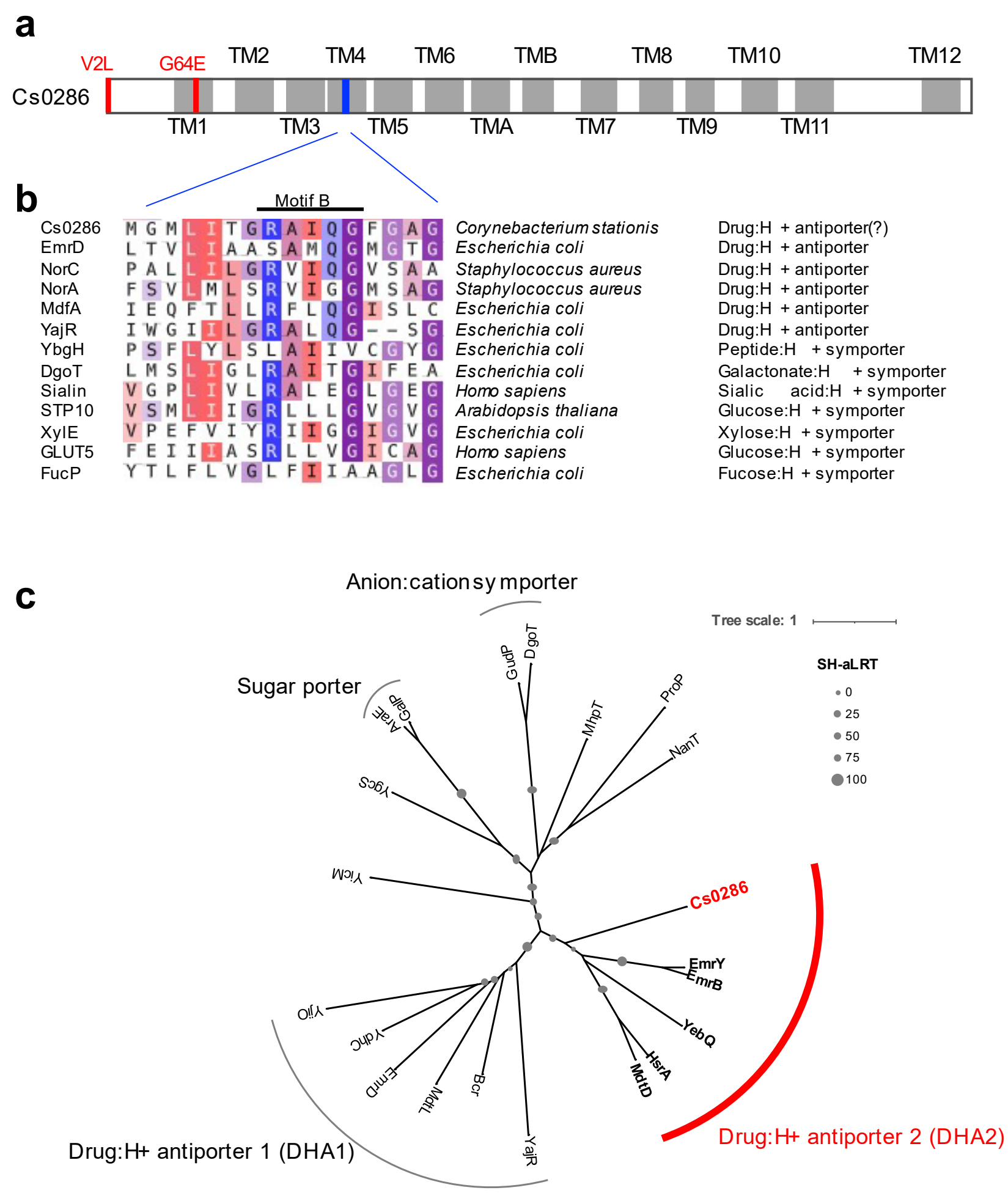

Fig. S3 Cs0286 is a major facilitator superfamily (MFS) transporter

(a) Protein structure of Cs0286. Transmembrane regions are colored gray.

(b) Alignment of sequences around Motif B in various MFS transporters.

(c) Maximum-likelihood tree of the Cs0286 transporter and its *E. coli* homologs. The tree was built using IQ-TREE, and the confidence levels with Shimohira-hasegawa approximate likelihood ratio test (SH-aLRT) are represented by the size of the circles on the nodes.

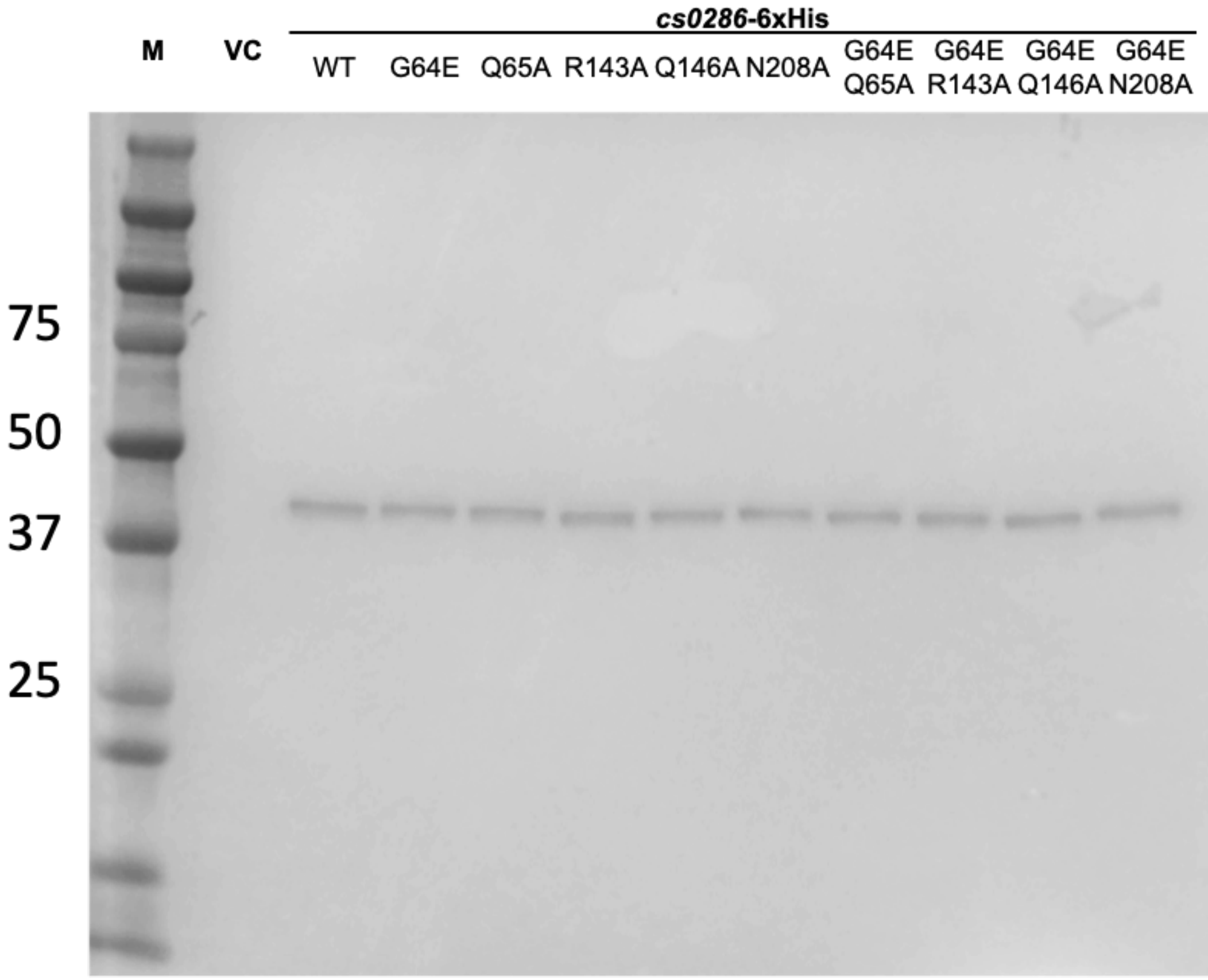

Fig. S4 Protein expression levels of cs0286 mutants.

His-tag fused cs0286 and its mutants were expressed and detected by Western blotting. One representative result of the three independent experiments is shown. M, marker; VC, vector control.

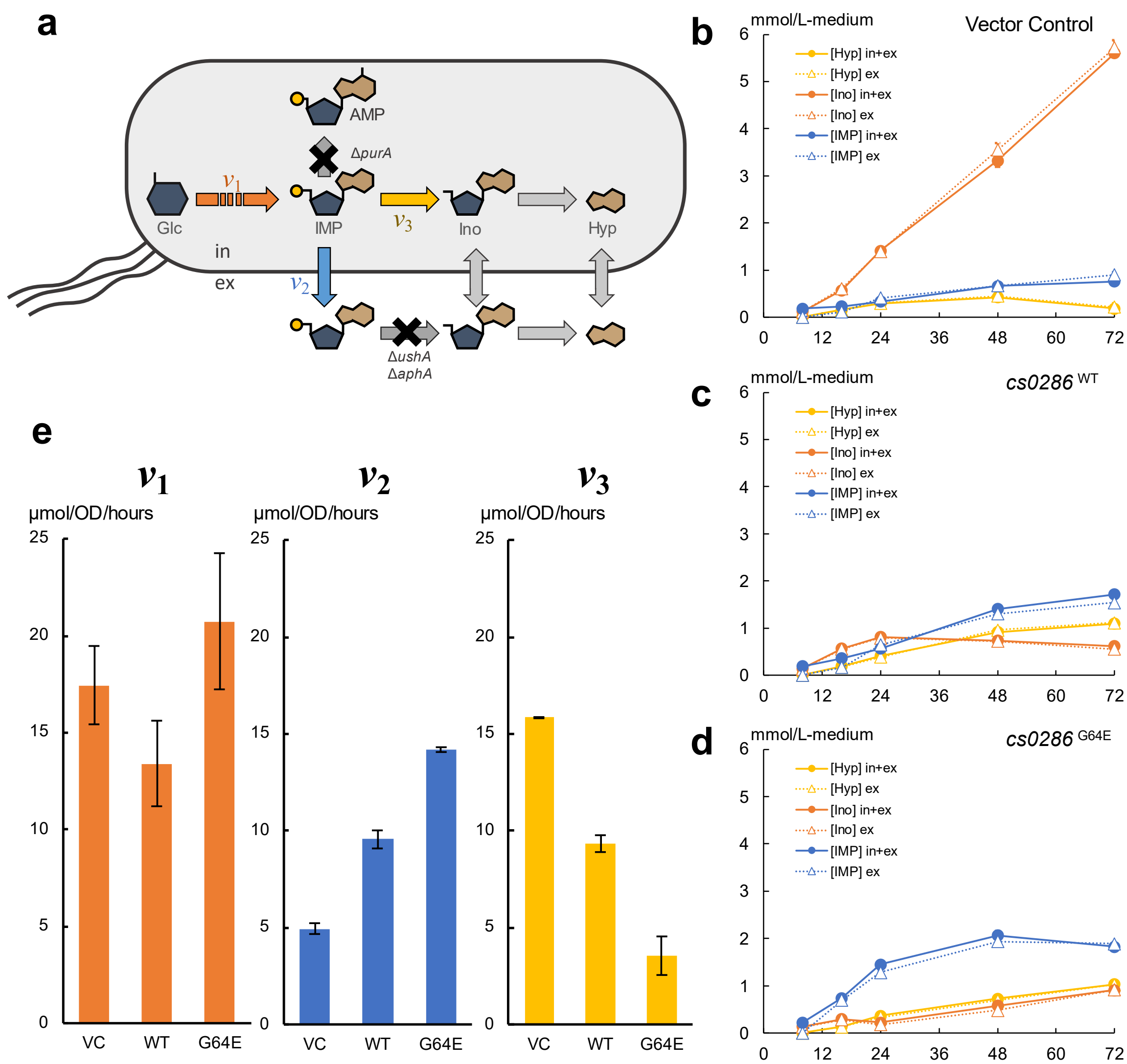

Fig. S5 The kinetics of IMP production.

(a) Metabolic pathway of IMP production in I-9*ushAaphA*/pMWKQ strain. Ino, Inosine; Hyp, Hypoxanthine.

(b-d) Extracellular (ex) and extracellular–intracellular total (in+ex) purine nucleotide levels of IMP-producing cultures with vector control (b), wild-type *cs0286* expressing strain (c). and *cs0286* $^{\mathrm{G64E}}$ expressing strain (d). The extracellular IMP data (dotted blue, [IMP]ex) are reprinted in the main Fig. 2b. Means of three independent experiments are shown and the bars represent standard errors.

(e) Rates of *de novo* purine nucleotide synthesis (v_1), IMP secretion (v_2), and IMP degradation (v_3) were calculated using 16 and 24 h data in panels b to d. Bars represent standard errors.

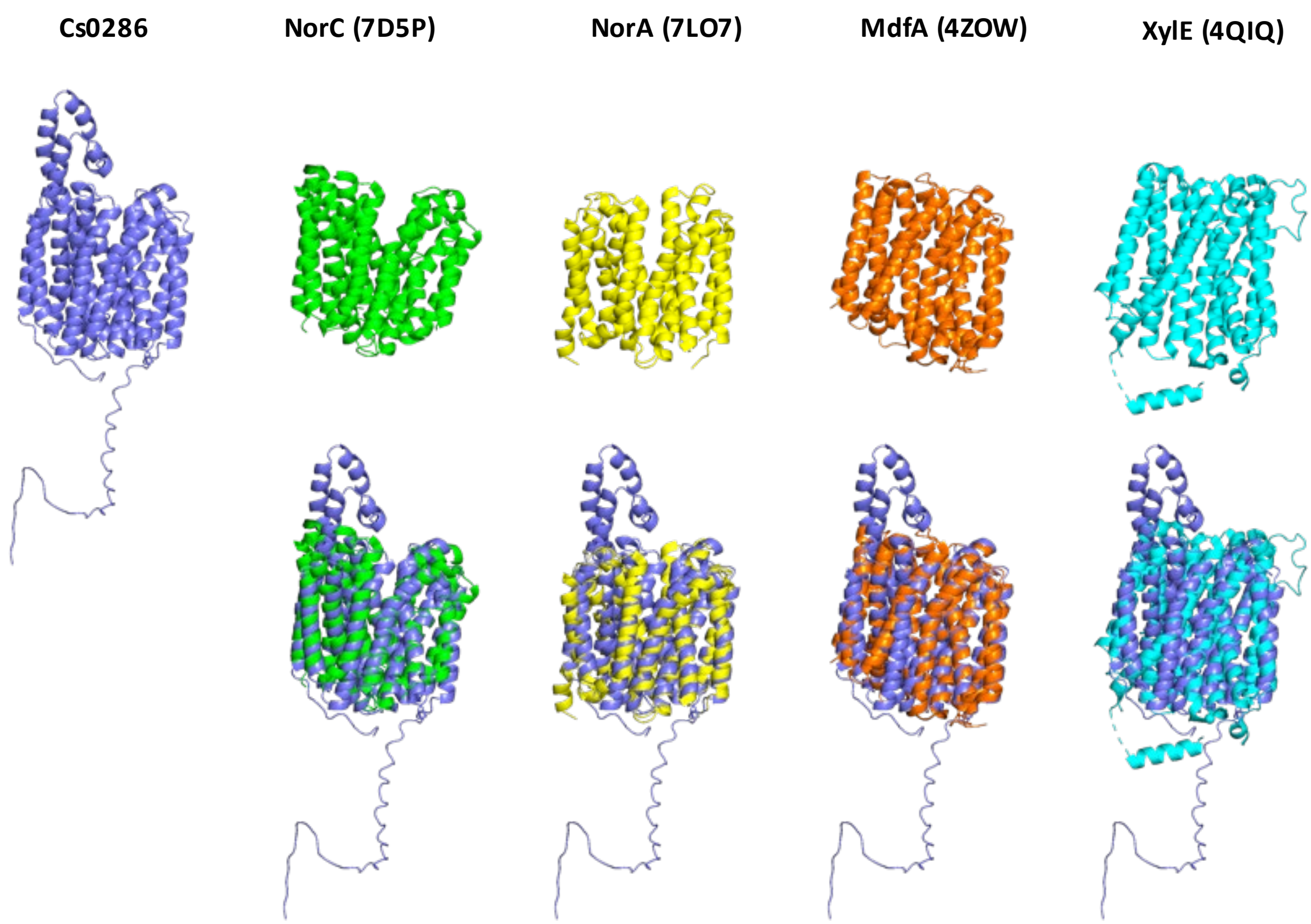

Fig. S6 Structure comparison with other MFS transporters.

Upper row: Cs0286 estimated structure and *Staphylococcus aureus* NorC (PDB: 7D5P, green), *S. aureus* NorA (PDB: 7LO7, yellow), *Escherichia coli* MdfA (PDB: 4ZOW, orange), and *E. coli* XylE (PDB: 4QIQ, blue). Cs0286 estimated structures aligned with each determined structure are shown in the lower row.

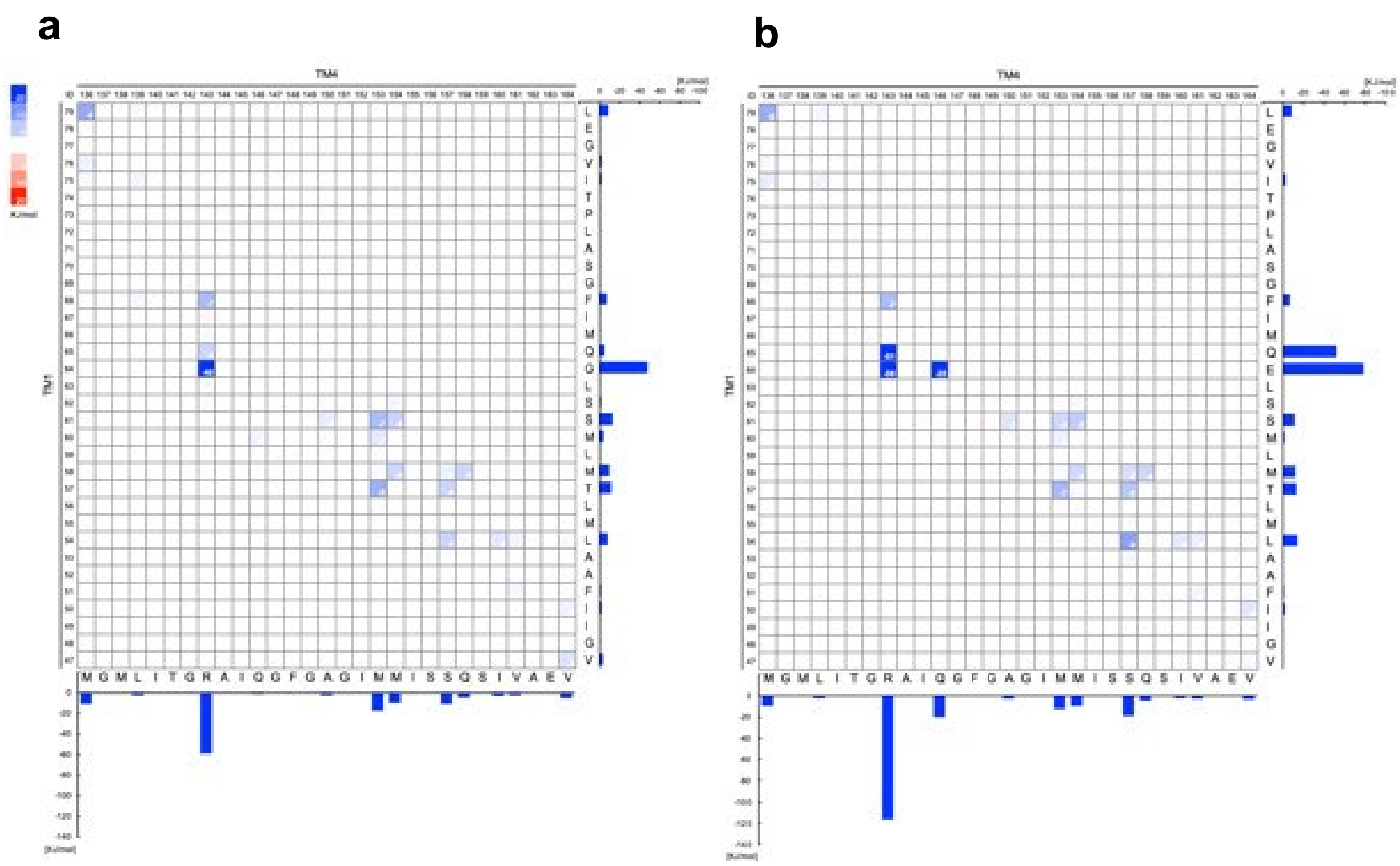

Fig. S7 Contribution of each residue in TM1–TM4 interactions.

Heatmap showing the interaction energies between each residue of TM1 and TM4 in the Cs0286 WT (a) and mutant (b). The cumulative interaction energies are represented by bar charts. The average values of three independent simulations are shown.

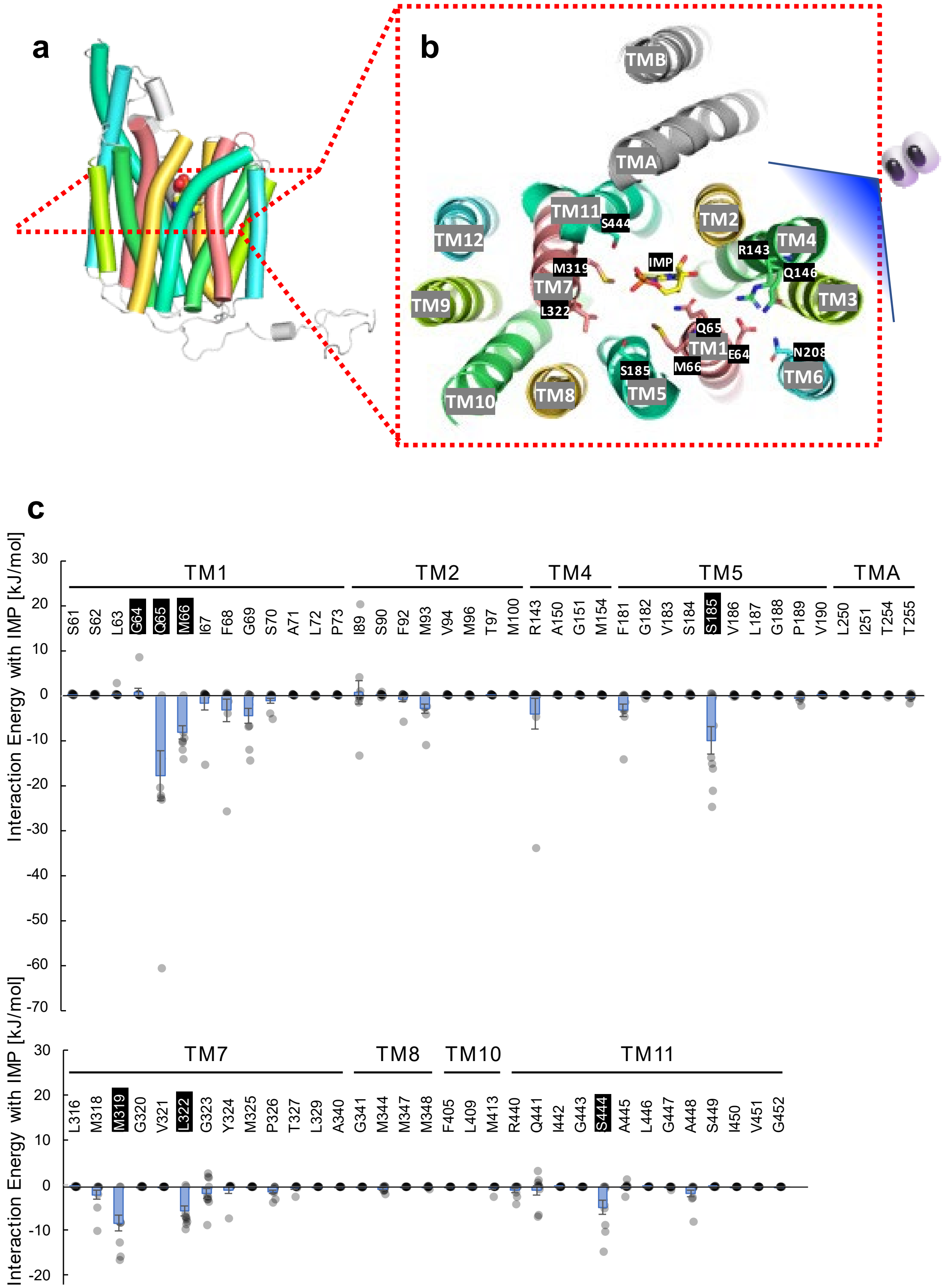

a
b
TMB
TMA
TM11
S444
TM2
TM4
R143
TM12
M319
IMP
Q146
TM7
TM9
TM3
L322
Q65
TM1
E64
N208
S185
M66
TM6
TM10
TM8
TM5
c
Interaction Energy with IMP [kJ/mol]
30
20
10
0
-10
-20
-30
-40
-50
-60
-70
TM1
TM2
TM4
TM5
TMA
S61 S62 L63 G64 Q65 M66 I67 F68 G69 S70 A71 L72 P73
I89 S90 F92 M93 V94 M96 T97 M100
R143 A150 G151 M154
F181 G182 V183 S184 S185 V186 L187 G188 P189 V190
L250 I251 T254 T255
Interaction Energy with IMP [kJ/mol]
30
20
10
0
-10
-20
TM7
TM8
TM10
TM11
L316 M318 M319 G320 V321 L322 G323 Y324 M325 P326 T327 L329 A340
G341 M344 M347 M348
F405 L409 M413
R440 Q441 I442 G443 S444 A445 L446 G447 A448 S449 I450 V451 G452

Fig. S8 Inosine mononucleotide (IMP)-interacting sites of Cs0286

(a,b) Representative image of the IMP-bound state of Cs0286 mutant. The viewpoint of Figure 5a, b and Movie 1 is shown in panel b.

(c) The interaction energies between IMP and the surrounding residues within 0.8 nm of IMP in the Cs0286 wild type. Each circle represents the average value over the last 100 ns of 10 independent simulations. The blue bars represent the average of 10 simulations. Error bars indicate standard error. Highlighted residues are shown in panel b.

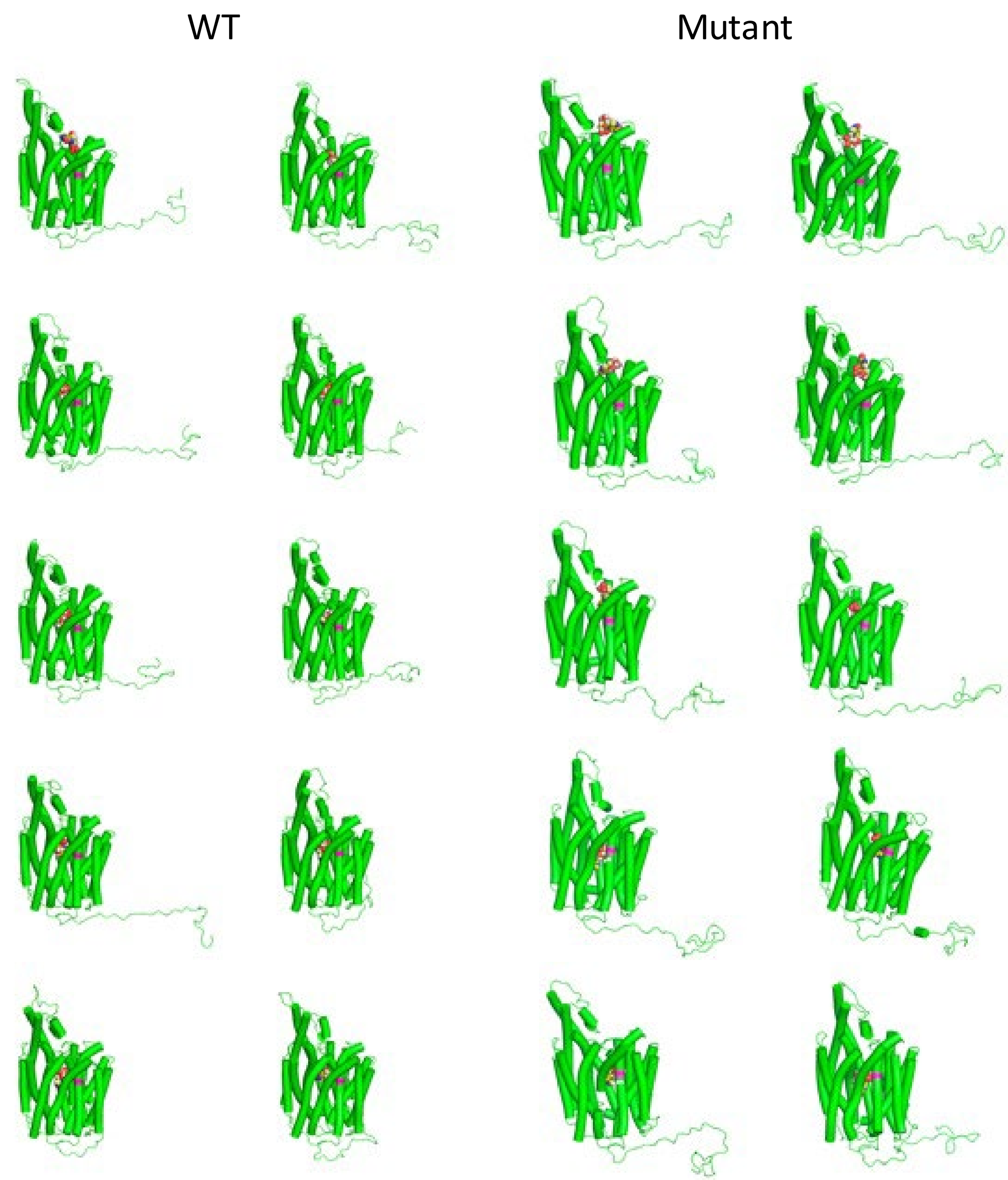

Fig. S9 Final structures of Cs0286 and its mutants in ten 340-ns independent molecular dynamics simulations in the presence of inosine mononucleotide (IMP).

IMPs are shown in the sphere model, and the positions of the G or E64 residues are colored in magenta.

Table S1. Details of the newly determined genome of *C. stationis* str. ATCC6872

	Size [bp]	CDS	tRNA	rRNA
ATCC6872	2,852,199	2,645	51	15
Liu *et al*., 2016	2,853,666	2,640	52	15

Table S2. Numbers of SNPs identified in analyzed strains

	Sequence depth	Filtered SNPs	Nonsynonymous mutations
KCCM10340	1195	427	253
KCCM10448	1373	625	381
KCCM10530	1617	624	381
KCCM10972	1871	619	377
KCCM10610	1796	985	614

SNP, single-nucleotide polymorphism.

Table S3. Annotations of convergently mutated transporters

Gene ID	Annotation	Mutations in XMP producers	Mutations in IMP producers
cs0286	Multidrug resistance protein 3	T43I	V2L G64E
cs0510	Putative multidrug-efflux transporter	A25V	G10D
cs0916	Putative pyrimidine permease RutG	A107V	G189D
cs0966	Putative FMN/FAD exporter YeeO	G160D	G355D
cs2429	Na(+)/H(+) antiporter subunit A	G72D	T765I P806S

IMP, inosine mononucleotide; XMP, xanthosine monophosphate

Table S4. Primers used for this study

#ID	Sequence
1	5′-CGTTGTAAAACGACGGCCAGTGAATTCTTATGCCTTTGCGCTGGCGTC-3′
2	5′-CAGGAGAATAACATGCTAGCTAAAAACTCCACC-3′
3	5′-GGTGGAGTTTTTAGCTAGCATGTTATTCTCCTG-3′
4	5′-GATGAGCTCTTTGGAGCAGATGATTTTCGGTTC-3′
5	5′-CCGAAAATCATCTGCTCCAAAGAGCTCATCAGC-3′
6	5′-CCTGGATTGCAGCTCCGGTGATCAGCATGC-3′
7	5′-GATCACCGGAGCTGCAATCCAGGGCTTCGG-3′
8	5′-CACCGAAGCCCGCGATTGCACGTCCGGTGATCAGCATGCC-3′
9	5′-ACGTGCAATCGCGGGCTTCGGTGCCGGCATCATGATGATT-3′
10	5′-CCAGTGGAATGGCGATCCACAGGCCCCAAC-3′
11	5′-CCTGTGGATCGCCATTCCACTGGGTCTGCT-3′
12	5′-CATGCTGGTTGGTGGCGGTAATATC-3′
13	5′-CATTTTTGCCAGCTCACCAATGCG-3′
14	5′-GTTGCGGTTGATATCGACGAACAGCG-3′
15	5′-GTTGGTTTCCATTTCGATGACCAGCTTG-3′
16	5′-CGTTGTAAAACGACGGCCAGTGAATTCTTATGCCTTTGCGCTGGCGTC-3′
17	5′-CAGGAGAATAACATGCTAGCTAAAAACTCCACC-3′
18	5′-GGTGGAGTTTTTAGCTAGCATGTTATTCTCCTG-3′
19	5′-GATGAGCTCTTTGGAGCAGATGATTTTCGGTTC-3′
20	5′-CCGAAAATCATCTGCTCCAAAGAGCTCATCAGC-3′
21	5′-CCTGGATTGCAGCTCCGGTGATCAGCATGC-3′
22	5′-GATCACCGGAGCTGCAATCCAGGGCTTCGG-3′
23	5′-CACCGAAGCCCGCGATTGCACGTCCGGTGATCAGCATGCC-3′
24	5′-ACGTGCAATCGCGGGCTTCGGTGCCGGCATCATGATGATT-3′
25	5′-CCAGTGGAATGGCGATCCACAGGCCCCAAC-3′
26	5’- TAAGAATTCACTGGCCGTCG -3’
27	5’-TTCGATGGTTTCCTTCAAGC-3’

Movie S1 (separate file). Representative movie of inosine mononucleotide detachment from the Q65 residue
